# Temperature dependent viral tropism: understanding viral seasonality and pathogenicity as applied to the avoidance and treatment of endemic viral respiratory illnesses

**DOI:** 10.1002/rmv.2241

**Published:** 2021-05-03

**Authors:** Patrick D. Shaw Stewart, Julia L. Bach

**Affiliations:** ^1^ Douglas Instruments Ltd. Hungerford Berkshire UK; ^2^ Amgen Inc. Thousand Oaks California USA

**Keywords:** Covid‐19, influenza, pathogenicity, seasonality, SARS‐CoV‐2, temperature sensitivity, viral respiratory illness

## Abstract

This review seeks to explain three features of viral respiratory illnesses that have perplexed generations of virologists: (1) the seasonal timing of respiratory illness and the rapid response of outbreaks to weather, specifically temperature; (2) the common viruses causing respiratory illness worldwide, including year‐round disease in the Tropics; (3) the rapid arrival and termination of epidemics caused by influenza and other viruses. The inadequacy of the popular explanations of seasonality is discussed, and a simple hypothesis is proposed, called temperature dependent viral tropism (TDVT), that is compatible with the above features of respiratory illness. TDVT notes that viruses can spread more effectively if they *moderate* their pathogenicity (thereby maintaining host mobility) and suggests that endemic respiratory viruses accomplish this by developing thermal sensitivity within a range that supports organ‐specific viral tropism within the human body, whereby they replicate most rapidly at *temperatures below body temperature*. This can confine them to the upper respiratory tract and allow them to avoid infecting the lungs, heart, gut etc. Biochemical and tissue‐culture studies show that ‘wild’ respiratory viruses show such natural thermal sensitivity. The typical early autumn surge of colds and the occurrence of respiratory illness in the Tropics year‐round at intermediate levels are explained by the tendency for strains to adapt their thermal sensitivity to their local climate and season. TDVT has important practical implications for preventing and treating respiratory illness including Covid‐19. It is testable with many options for experiments to increase our understanding of viral seasonality and pathogenicity.

**Abbreviations**
TDVT: temperature dependent viral tropism
ts: thermally sensitive

## 1 DEFINITION OF TERMS

In this review, ‘dormant’ means biochemically inactive, or active at low levels. It may imply no viral replication, or low levels of replication. As discussed below, dormant virions may be invisible to the host immune system. (The word ‘latent’ is avoided because this refers to retroviruses and herpesviruses whose genomes persist within the host, from which they can subsequently reactivate, which are not considered here.) The locations of dormant viruses in the respiratory tract (in, on or between cells) is unknown, and may vary.

A ‘mild’, ‘attenuated’ or ‘less pathogenic’ respiratory virus is one that is *more thermally sensitive (ts)*. Such viruses possess biochemistry that is most active at temperatures *below* normal body temperature. A ‘virulent’ or ‘highly pathogenic’ virus is one that is *less ts,* or active at both body temperature and below. A highly pathogenic virus is likely to cause respiratory illness at any ambient temperature. A mild respiratory virus is likely to cause respiratory illness only at low ambient temperature. For a mild respiratory virus, the ‘permissive’ temperature is the temperature of the nose and throat, while the temperature of the lungs is ‘restrictive’. For a highly pathogenic virus the temperature of all parts of the body is permissive.

## 2 INTRODUCTION: CONVENTIONAL EXPLANATIONS OF THE SEASONALITY OF VIRAL ILLNESS, AND THEIR PROBLEMS

Scientists have for decades recognised the tendency of viral illnesses to worsen during predictable seasons.^1^, ^2^ This review focuses on four features of the respiratory viral illness seasonality that lack rigorous and convincing scientific explanations:

- Seasonal timing and weather influence.
- Common viruses worldwide.
- Rapid arrival and termination.

### 2.1 Seasonal timing and weather influence

**FIGURE 1:**
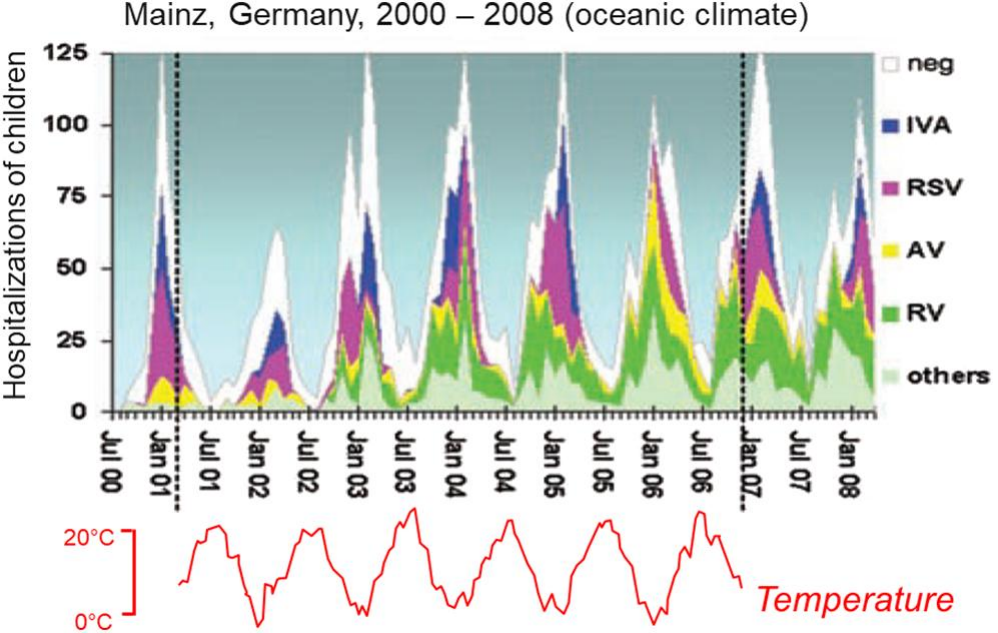
An example of winter seasonality: the viruses that caused respiratory infections in hospitalised children in Mainz, Germany, which has an oceanic climate, 2000–2008. The timing of illness caused by particular viral species is variable. For example, RSV hospitalizations mainly occurred before 1 January in the winter of 2002/03, but after 1 January from 2005 onwards. Nevertheless, the maximum for all hospitalizations was normally around February, and the minimum around August. Adapted from du Prel JB et al. Clin Infect Dis 2009;49(6):861–8^3^

**FIGURE 2:**
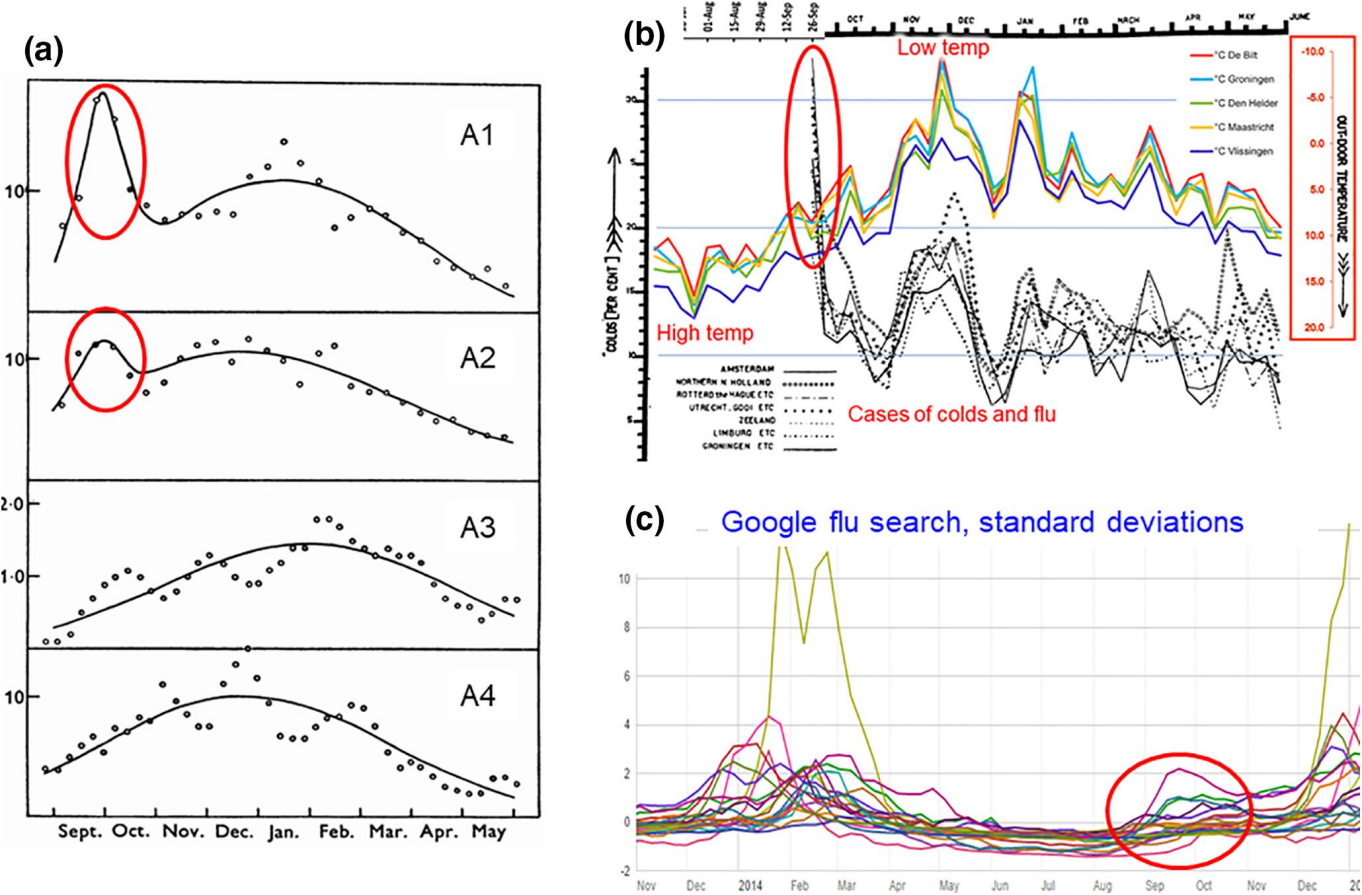
Four datasets that show an ‘autumn surge’ in colds (red ovals). (a) Graphs show the average yearly incidence of colds for each period of 10 days +/− 1 day^13^ A1: London, 1951–1955; A2: Newcastle, 1951–1957; A3: Cirencester, 1954–1956; A4: Chalke valley, 1948–1949. For London and Newcastle the number at the end of September was greater than or equal to the number in midwinter. In Cirencester, A3, and Chalke valley, A4, smaller autumn increases were seen. (b) At the beginning of van Loghem's study in September 1925 one third of respondents in Amsterdam had colds, with high numbers in other regions (red oval).^14^ For more information see Figure 4. (c) In September 2014 Google ‘flu search’ activity increased in most countries in the Northern Hemisphere (red oval). For more information see Figure 3 (a) is adapted from Epidemiology & Infection, 1965 Sep; 63 (3):427–39^13^; (b) from Epidemiology & Infection, 1928 Aug; 28 (1):33–54^14^; (c) Retrieved August 2015

The seasonal occurrence of viral respiratory illness in temperate regions, with more illness during colder seasons (Figure 1), including a surge in respiratory illness that frequently appears in early autumn (Figure 2), demands scientific explanation. The shared winter seasonality of so many unrelated viral species is extraordinary.^3^, ^4^ The diverse characteristics of some important human respiratory viruses that cause illness with winter seasonality are shown in Table 1.

**TABLE 1:** Characteristics of common winter season respiratory viruses (exception: parainfluenza type 3)

Family	Viruses causing human respiratory illness	Primary genetic material	Replication site in the cell	Presence of lipid envelope	Virion shape
Adenoviridae	Adenovirus	Double‐stranded DNA	Nucleus	No envelope	Icosahedral
Coronaviridae	Coronavirus, SARS virus, SARS‐CoV‐2	Positive‐sense single‐stranded RNA	Cytoplasm	Enveloped	Spherical with projections
Herpesviridae	Varicella zoster virus (chickenpox)	Double‐stranded DNA	Nucleus	Enveloped	Icosahedral
Orthomyxoviridae	Influenza virus	Negative‐sense single‐stranded RNA	Nucleus	Enveloped	Spherical or filamentous
Paramyxoviridae	Measles, mumps, parainfluenza, respiratory syncytial viruses, human metapneumovirus	Negative‐sense single‐stranded RNA	Cytoplasm	Enveloped	Spherical or variable
Picornaviridae	Rhinovirus	Positive‐sense single‐stranded RNA	Cytoplasm	No envelope	Icosahedral
Togaviridae	Rubella virus	Positive‐sense single‐stranded RNA	Cytoplasm	Enveloped	Icosahedral

The most popular explanations of the winter seasonality of respiratory viruses fall into three categories, designated here as follows^a^: E1, **crowding** of human hosts in winter encourages transmission; E2, ambient conditions allow respiratory viruses to **survive** outside the body longer in cold and dry conditions; E3, the **immune defences** of human hosts are weaker during the winter months. These explanations are important, but do not describe the main drivers of epidemic timing.

**FIGURE 3:**
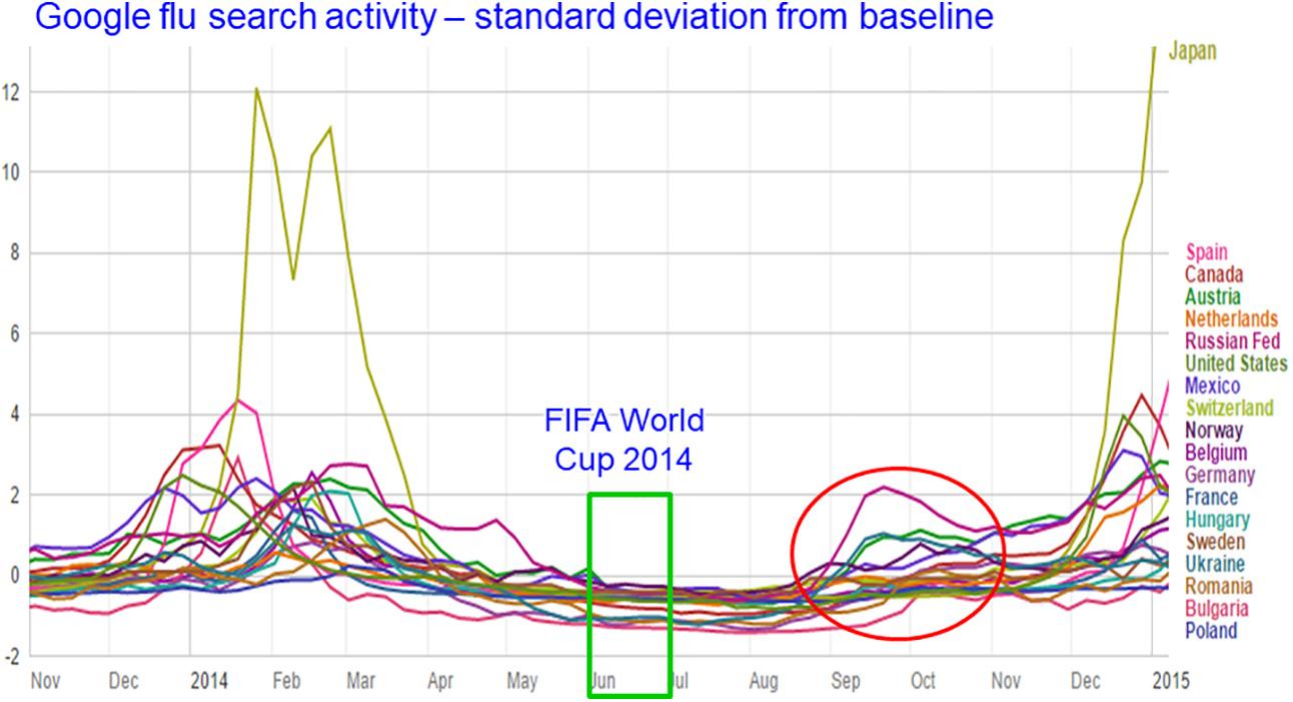
Google ‘flu search’ activity during FIFA 2014. Google flu search measured the number of people who search for terms related to colds and flu using the Google search engine. During major sporting competitions people often crowd together to watch the events. During the 2014 FIFA football World Cup, in the 18 countries in the Northern Hemisphere where Google reported flu search activity, there was no increase in the number of Google searches for related terms. This suggests that the winter epidemics displayed are not mainly driven by crowding (E1). Notice the epidemics of colds in September 2014 (red oval). Autumn epidemics are common when the temperature dips at the end of summer (Figure 2)

**Crowding** (E1) would predict that summer sporting events and festivals would be associated with increased respiratory illness, which is not the case. Tamerius et al. showed this lack of association for influenza.^2^ Figure 3 shows that ‘Google flu search’ did not increase in any country in the Northern Hemisphere during the FIFA 2014 football World Cup,^b^ despite spectators and fans packing into crowded bars, restaurants and homes to view World Cup matches. School vacations are not well‐correlated with respiratory illness. For example, children are on vacation in Singapore in December, which coincides with the yearly *peak* of influenza.^2^ The crowding hypothesis E1 would also predict that colds and flu would be much more prevalent in large cities than in the countryside, which is not the case.^5^

**Survival** (E2) focuses on the suggestion that cold and dry conditions increase the survival of the virus outside the body, thereby encouraging transmission. The corollary is that E2 would also predict that colds and flu would be rare in warm, wet or humid weather. However, in tropical locations that have rainy seasons, colds and flu are most prevalent during those seasons, when humidity is likely to be high.^2^, ^6^ It has been suggested that air conditioning in the Tropics may enhance viral survival by cooling and drying indoor air.^7^ However, in some Tropical locations such as Fortaleza (Brazil), temperatures during the rainy season are slightly lower than in the dry months, so air‐conditioning use is unlikely to be higher, yet colds and flu are much more prevalent in the rainy season.^2^, ^6^ Also, World Health Organization reports show that influenza was common year‐round in the Tropics during the period 1964–1975, a time when most people in the Tropics did not have air conditioning.^8^

The effect of humidity on virus survival can be examined experimentally. Table 2 summarises studies where viruses were introduced as aerosols into rotating drums with controlled humidity. The survival of some viruses, including influenza A, improved in dry conditions, but when influenza A viruses were supplemented with material from the apical surface of human airway epithelial cells they remained equally infectious when relative humidity was varied from 23% to 98%.^9^ Moreover, other viruses, including rhinovirus‐14, rhinovirus‐16, and adenovirus, were more stable in humid conditions.^10^, ^11^, ^12^ The illnesses caused by these viruses are nevertheless seasonal in temperate regions in the cold, dry air of winter, with virtually all other viral respiratory illnesses. None of this information supports the idea that it is the dry conditions of winter specifically that are ideal for the survival of respiratory viruses and thus explain the timing of cold and flu seasons.

**TABLE 2:** Laboratory studies of the survival of respiratory viruses in aerosols, including several that are more stable in humid air

First author	Year	Ref.	Respiratory virus	Presence of lipid envelope	Association between virus survival and relative humidity
Harper	1961	103	Influenza A	Enveloped	Anti‐correlated
Songer	1967	104	Newcastle disease virus	Enveloped	Anti‐correlated
Songer	1967	104	Infectious bovine rhinotracheitis	Enveloped	Correlated
Akers	1968	105	Mengovirus 37A	Non‐enveloped	Correlated
Hugh‐Jones	1973	106	Newcastle disease virus	Enveloped	Correlated
Schaffer	1976	107	Influenza A	Enveloped	Least stable at 50% RH
Elazhary	1979	108	Infectious bovine rhinotracheitis	Enveloped	Variable, usually anti‐correlated
Elazhary	1979	109	Bovine adenovirus 3	Non‐enveloped	Correlated
Elazhary	1979	12	Bovine parainfluenza type 3	Enveloped	Anti‐correlated
Karim	1985	10	Rhinovirus‐14	Non‐enveloped	Correlated
Ijaz	1985	110	Human coronavirus 229E	Enveloped	Peaks at 50% RH (20°C)
Schoenbaum	1990	111	Pseudorabies	Enveloped	Peaks at 55% RH
Kormuth	2018	9	Pandemic influenza A (H1N1)	Enveloped	Viruses supplemented with material from airway cells were equally infectious at all humidities
Schuit	2020	112	SARS‐CoV‐2	Enveloped	No effect in darkness, variable in sunlight
Niazi	2021	11	Rhinovirus‐16	Non‐enveloped	Lowest survival around 50% (static RH). 97% of the virus was lost during the transition from 80% to 50% RH.

Abbreviation: RH, Relative Humidity.

**FIGURE 4:**
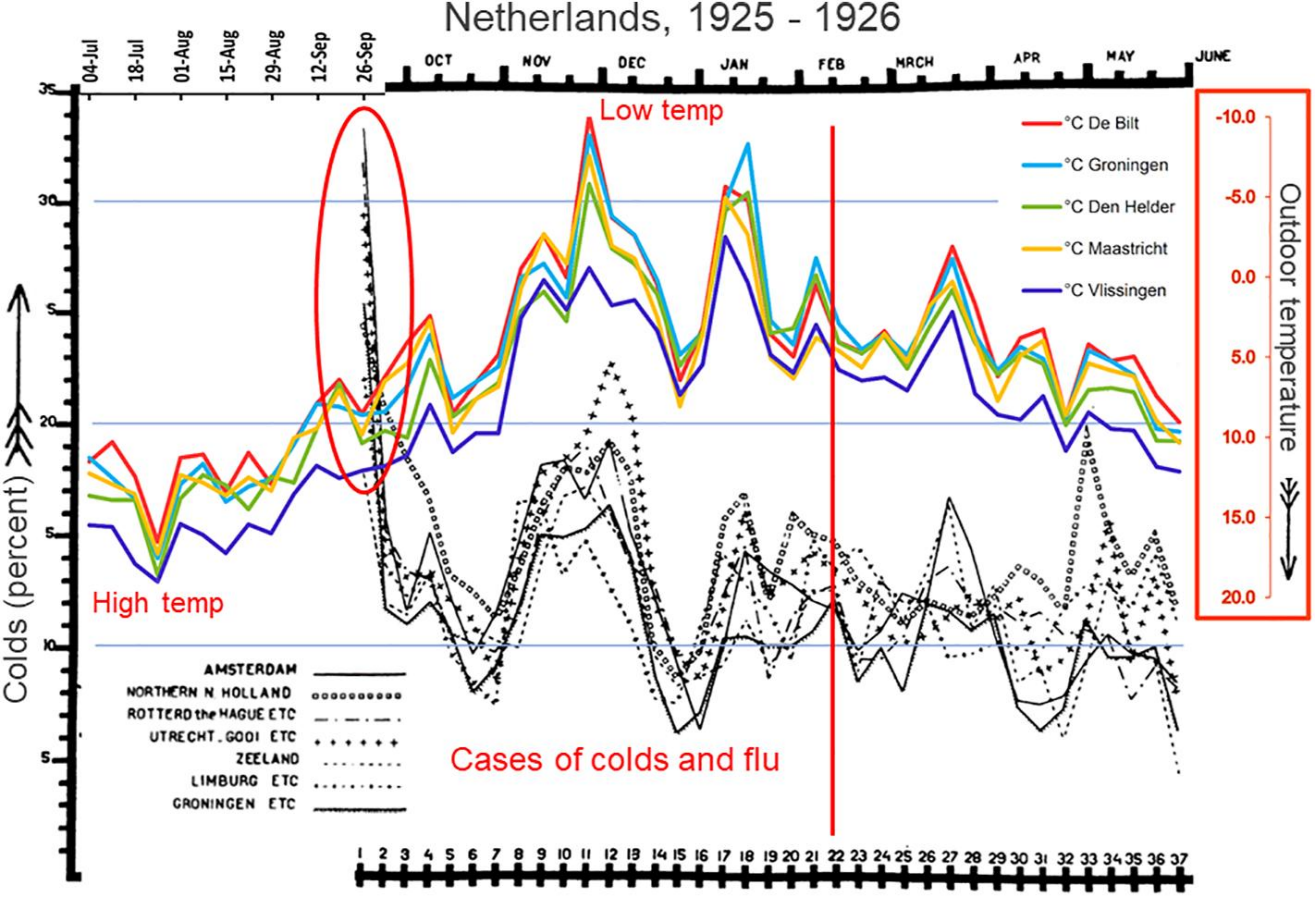
Graph II from van Loghem's report on the epidemiology of respiratory illnesses in the Netherlands in the winter of 1925/26, superimposed with ambient temperature from five Dutch weather stations (coloured lines), temperature scale inverted (lowest temperatures at the top). The highest number of illnesses in the whole study was at the beginning, in September 1925. There is also an extraordinary correlation between ambient temperature, and colds and flu throughout the Netherlands. In 1925 colds and flu arrived in all areas of the Netherlands almost simultaneously, with illnesses synchronised to within 1 – 2 weeks at some points. After the red line the relationship between temperature and colds became less clear. Adapted from Epidemiology and Infection 28 (01), 33–54^14^

Studies also show that the onset of respiratory illness outbreaks in response to temperature change is too rapid to be caused by *increased transmission* via *conditions favouring virus survival*. A study by Lidwell et al. examined the effect of weather on colds that were recorded during 5000 person‐years in two cities in the UK during the 1950s^13^ The strongest correlation was between colds and *temperatures recorded only two to three days before* the onset of colds, with a correlation coefficient of −0.162.^13^ Van Loghem carried out a similar large‐scale study in the Netherlands during the winter of 1925/26,^14^ and found a similar very rapid response of colds to temperature falls, too fast to be caused by increased transmission (Figure 4). Hajat et al. found an association between low temperatures and an increase in lower respiratory tract consultations in 16 urban locations in the UK.^15^ The authors also tracked relative humidity but found only ‘a weak non‐linear relationship’ with health outcomes, indicating that increased virus survival with low humidity was likely not a cause. Fluctuations in ambient temperature are what appear to be crucial for most respiratory viruses, with rapid starts to outbreaks that cannot be accounted for by increased transmission.

A recent review of Covid‐19 data subscribes to the virus survival explanation, but mostly focuses on absolute temperature and humidity, not weather *changes*, and concludes that much of the data is confounded with public policies implemented in the reaction to the pandemic.^16^ However, Covid‐19 outbreaks in many areas have followed regional weather patterns. The autumn 2020 wave of Covid‐19 in the USA began in the Midwestern states,^17^, ^18^ which experienced two severe, record‐breaking cold snaps in September and October 2020.^19^ SARS‐CoV‐2 data will continue to stream in, but it (already) appears to be affected by weather changes too rapid to be accounted for by increased transmission.

The **immune defences** (E3) explanation assumes that the immune system does not function as well in the colder, darker conditions of winter in temperate regions. However, Paynter et al. found that vaccines were slightly more effective in winter than in summer.^20^ An early study on an isolated tropical island found that colds increased in September when the temperature dropped by only 1°C–2°C, from around 24°C at night to around 22°C.^21^ It seems implausible that such a small temperature drop, starting at a relatively high temperature, could meaningfully depress human immunity. Experimental studies by Andrewes, and Dowling et al., suggested that immune defences are not made meaningfully weaker by experimental chilling of individuals (although these studies used ‘pedigree’ viral strains that may behave differently from wild‐type strains, discussed below).^22^, ^23^

**FIGURE 5:**
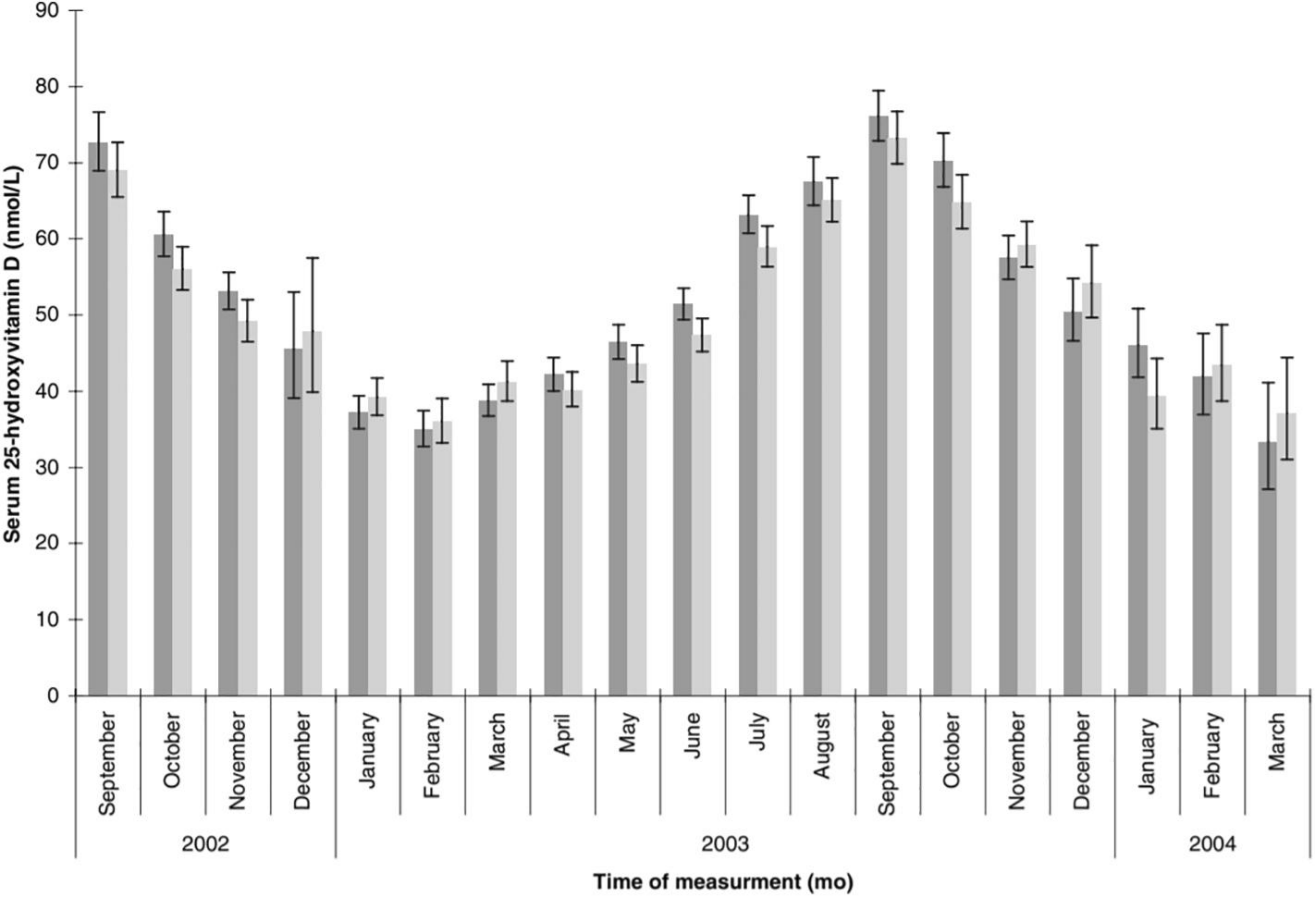
Monthly serum levels of vitamin D in men (dark grey) and women (light grey). Vitamin D levels change slowly over several months, with most increases and decreases being monotonic. Vitamin D levels were highest in September, which coincides with the frequent *surge* in colds during that month (figure 2). This figure was originally published in Am J Clin Nutr 2007;85:860–868^24^

Others propose that the winter seasonality of Covid‐19 and other viral illnesses is driven by vitamin D deficiency due to reduced sunlight during the winter months. However, vitamin D levels typically peak in September in temperate locations (Figure 5),^24^ which is when the autumn surge of respiratory illness arrives (Figure 2). Also, vitamin D levels change slowly, typically over a few months (Figure 5), whereas respiratory illness often follows temperature drops within two weeks (Figure 4). Lastly, colds and flu do not follow overcast weather, and do not decrease after sunny weather.^13^ Lack of vitamin D or other deficiencies brought on by insufficient sunshine may contribute to the prevalence of colds and flu in late winter and early spring,^c^ but they are unlikely to be the main drivers of winter seasonality.^25^, ^26^

### 2.2 Common viruses worldwide

**FIGURE 6:**
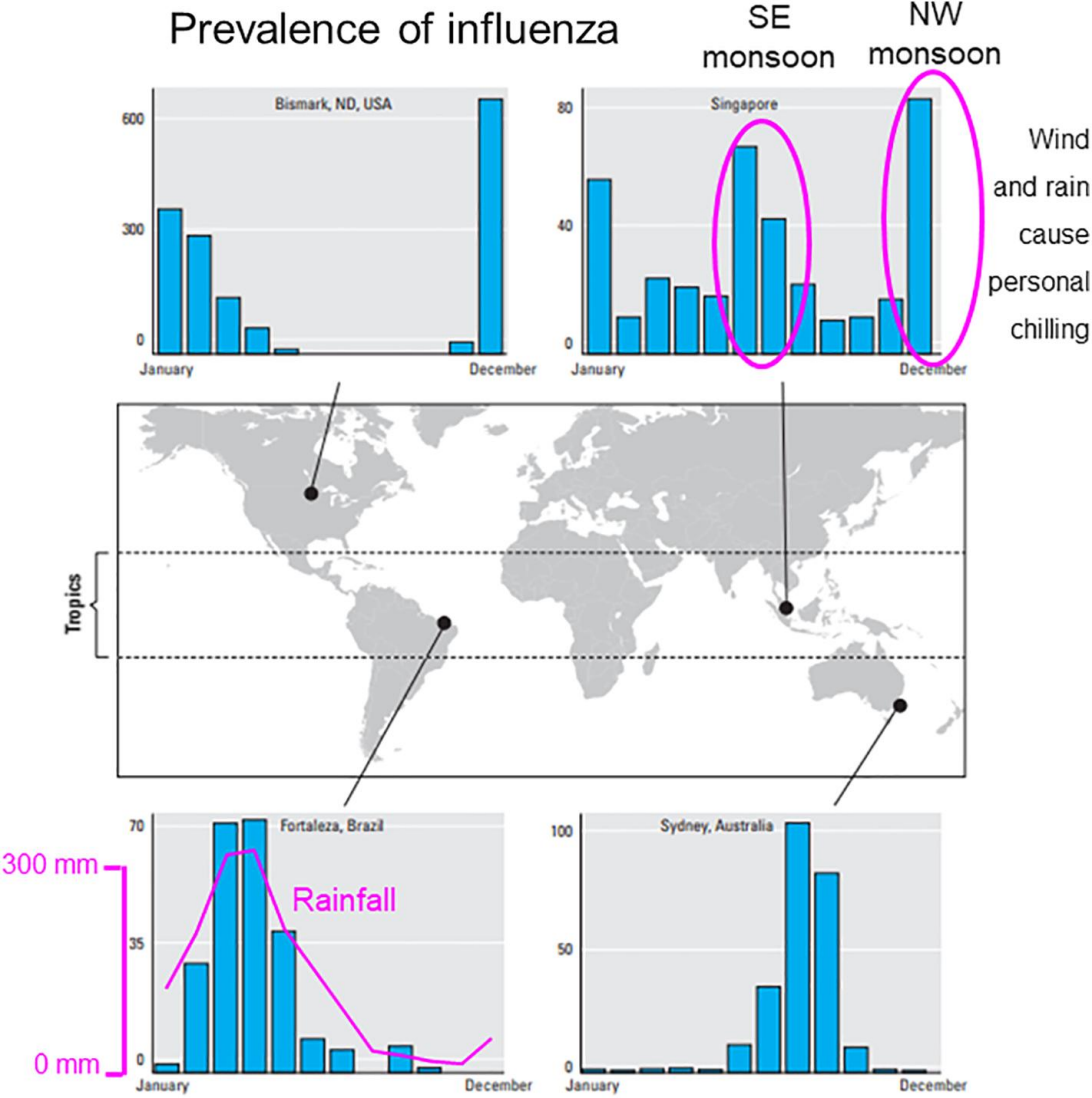
Seasonal patterns of influenza in four sites across several latitudes worldwide. In temperate regions epidemics normally occur during the winter months. Seasonal influenza activity in the Tropics is greatest during the rainy season. The bar charts indicate the average number of detected influenza isolates (*y*‐axis) over several years for Singapore, Fortaleza (Brazil), Bismarck (North Dakota, USA) and Sydney (Australia). Rainfall in Fortaleza and the two monsoons in Singapore are shown in purple. Adapted from Environmental health perspectives, 2011 Apr;119(4):439‐45^2^

Table 3 lists the viruses that most frequently caused hospitalization of children in three studies based at hospitals at different latitudes.^3^, ^4^, ^27^ There are striking similarities. Moreover, viral respiratory illnesses are rare in mid‐summer in temperate regions, but they usually occur throughout the year at moderate levels in the Tropics (sometimes with a surge in the rainy season^6^). For example, Figure 6 shows that influenza is almost absent during the summer in Northern USA and Sydney (Australia), but it is present year‐round in Singapore and during the rainy season in Fortaleza (Brazil). The same trends can be seen in the prevalence of influenza reported by the World Health Organization for the period 1964–1975.^8^

**TABLE 3:** Respiratory viruses that most frequently cause hospitalizations of children in temperate and tropical locations

City	Ref.	Climate	Most common respiratory viruses causing hospitalization of children
Mainz, Germany	3	Oceanic	Rhinovirus, RSV, influenza A, adenovirus
Buenos Aires, Argentina	4	Humid subtropical	RSV, influenza A, adenovirus, parainfluenza
Singapore	27	Tropical	RSV, parainfluenza, influenza A, adenovirus

Abbreviation: RSV, respiratory syncytial virus.

### 2.3 Rapid arrival and termination

**FIGURE 7:**
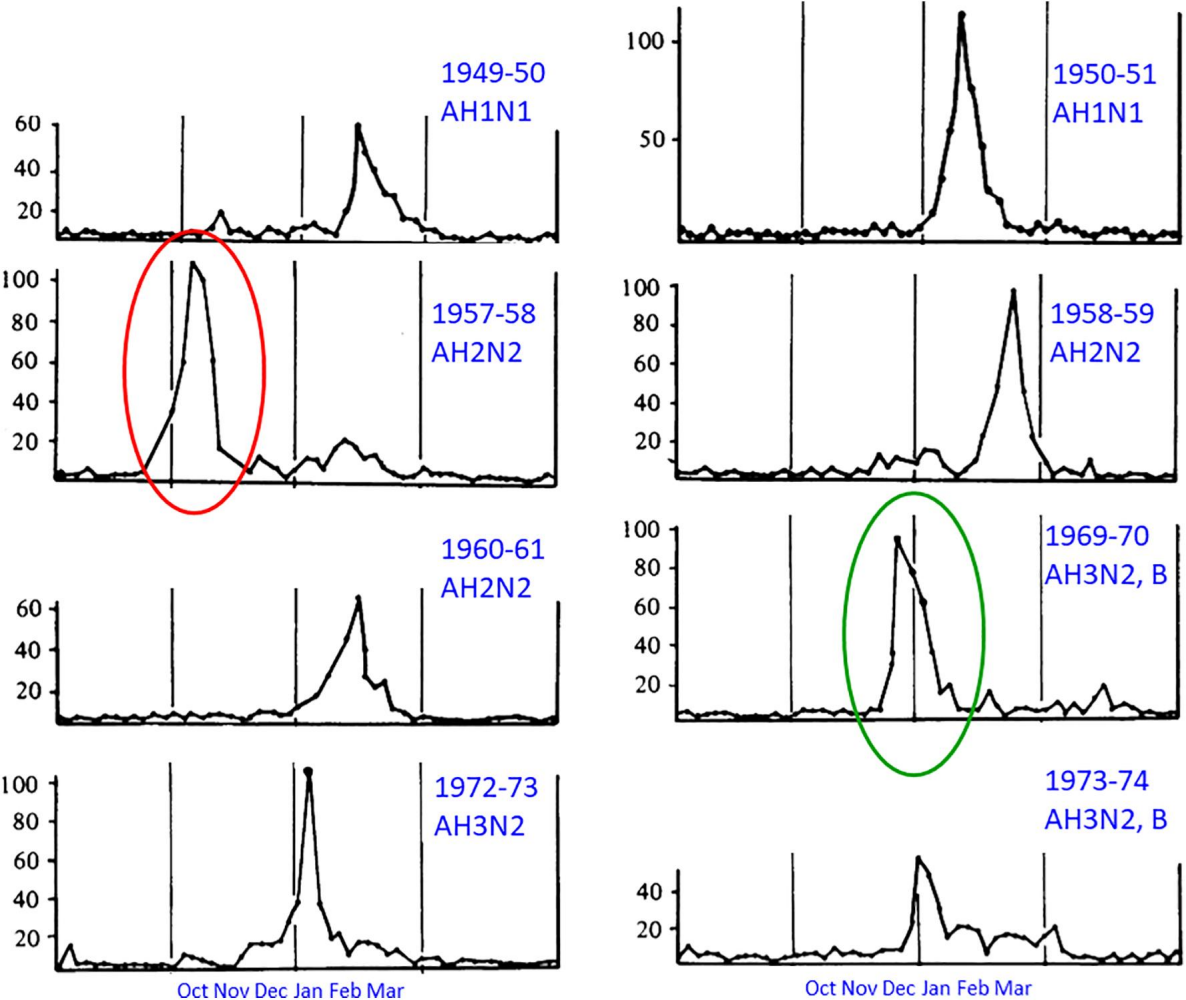
The eight largest influenza outbreaks in Cirencester, UK, 1946–1974. The graphs show the number of patients treated for acute febrile respiratory illness. Influenza epidemics sometimes arrive in just 2–3 weeks and terminate in a similar period. The red oval shows the arrival of Asian flu, green, the first major epidemic of Hong Kong flu (which arrived the previous year). Adapted from Epidemiology & Infection, 1981 Feb;86(1):35‐47^8^

**FIGURE 8:**
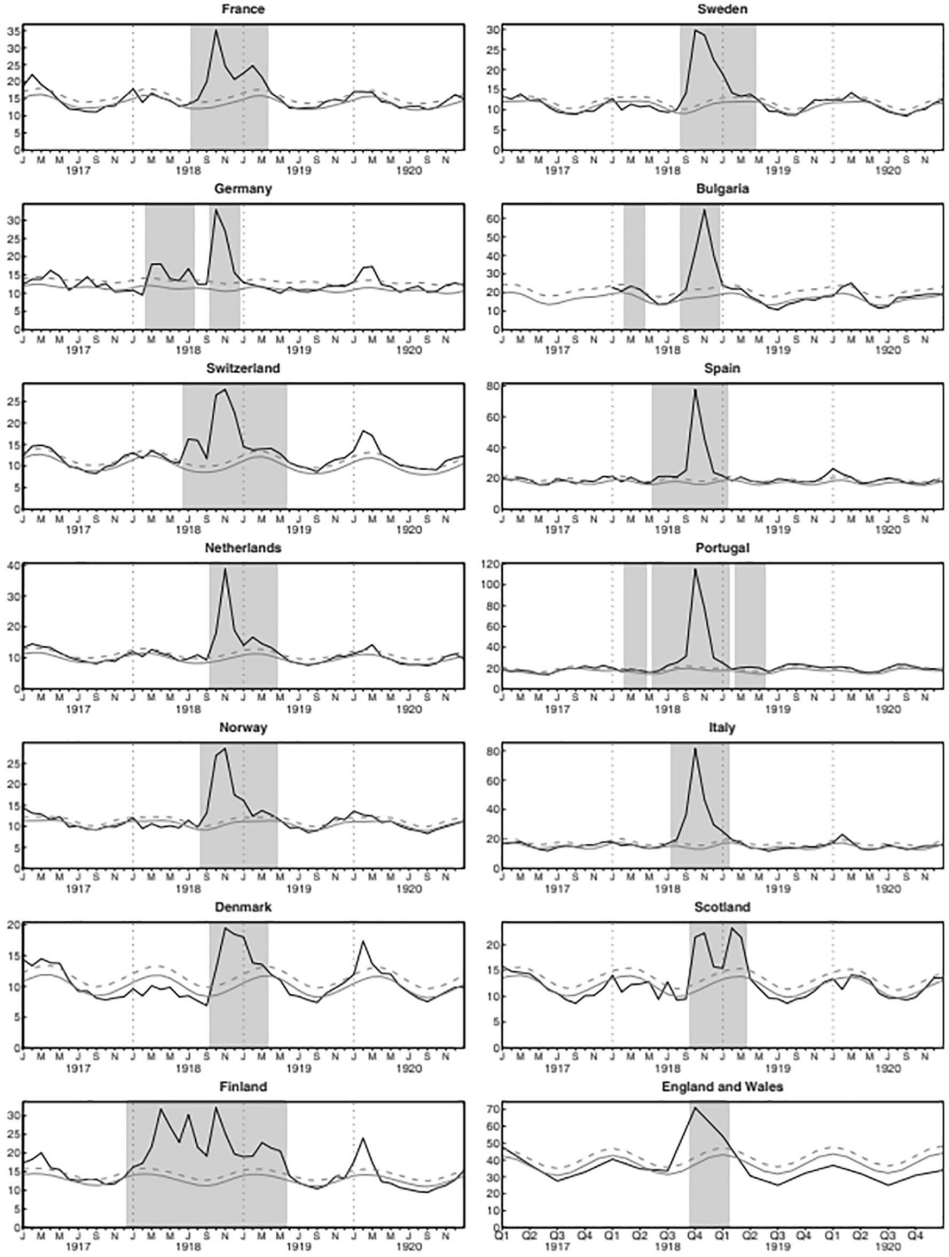
Mortality rate (black line) in 14 European countries during the Spanish Influenza epidemic, 1917–1920. The continuous grey line shows predicted mortality, the dashed grey line the pandemic threshold.^29^ Most countries experienced only one main peak, occurring in the same two‐month period. Epidemics the following winter were much smaller and only apparent in four countries. Mortality in Finland is difficult to interpret during and after the Finnish Civil War (January1918 to May 1918). A common explanation for the rapid termination of this epidemic is that almost the whole population was exposed to the virus, and that individuals either died or acquired immunity. However, recent studies have suggested that the extreme pathogenicity of Spanish flu arose from the unusual selective conditions that existed in the armed forces during World War I,^29^ and the disease's pathogenicity may have decreased when increased immunity in the population encouraged the natural selection of milder strains, as suggested by TDVT and the well‐known ‘trade‐off’ hypothesis. This figure was originally published in Influenza and Other Respiratory Viruses, 2009 May;3(3):99–106^29^

As characterized by Hope‐Simpson, an ‘explosive’ arrival and rapid termination is observed for many epidemics of influenza and other endemic respiratory viruses (Figure 7). Influenza frequently arrives simultaneously throughout large geographical areas, in isolated remote rural settings and nearby towns and cities, and even widely separated locations at similar latitudes.^5^, ^8^ Hope‐Simpson showed extraordinary synchronisation in influenza epidemics arriving in Cirencester (UK) and Prague between 1969 and 1974, including an antigenic shift from A/HK/68 to A/ENG/42/72 in 1972 and a transition to influenza B in 1973.^8^, ^28^ Figure 8 shows that during the Spanish Influenza epidemic of 1918/19 most European countries experienced a single peak of mortality during a two‐month window (October and November 1918), with winter mortality returning to normal levels in all but four countries in 1920.^29^ For Covid‐19, cases have rapidly decreased at unexpected times during the pandemic.^30^, ^31^ For example, the World Health Organization reported that at the end of January 2021 Covid‐19 cases declined in five of six world regions (the exception being tropical South‐East Asia) in spite of a lack of widespread immunity and limited vaccination.^32^

## 3 THE FOUR PREMISES OF TEMPERATURE DEPENDENT VIRAL TROPISM (TDVT)

**FIGURE 9:**
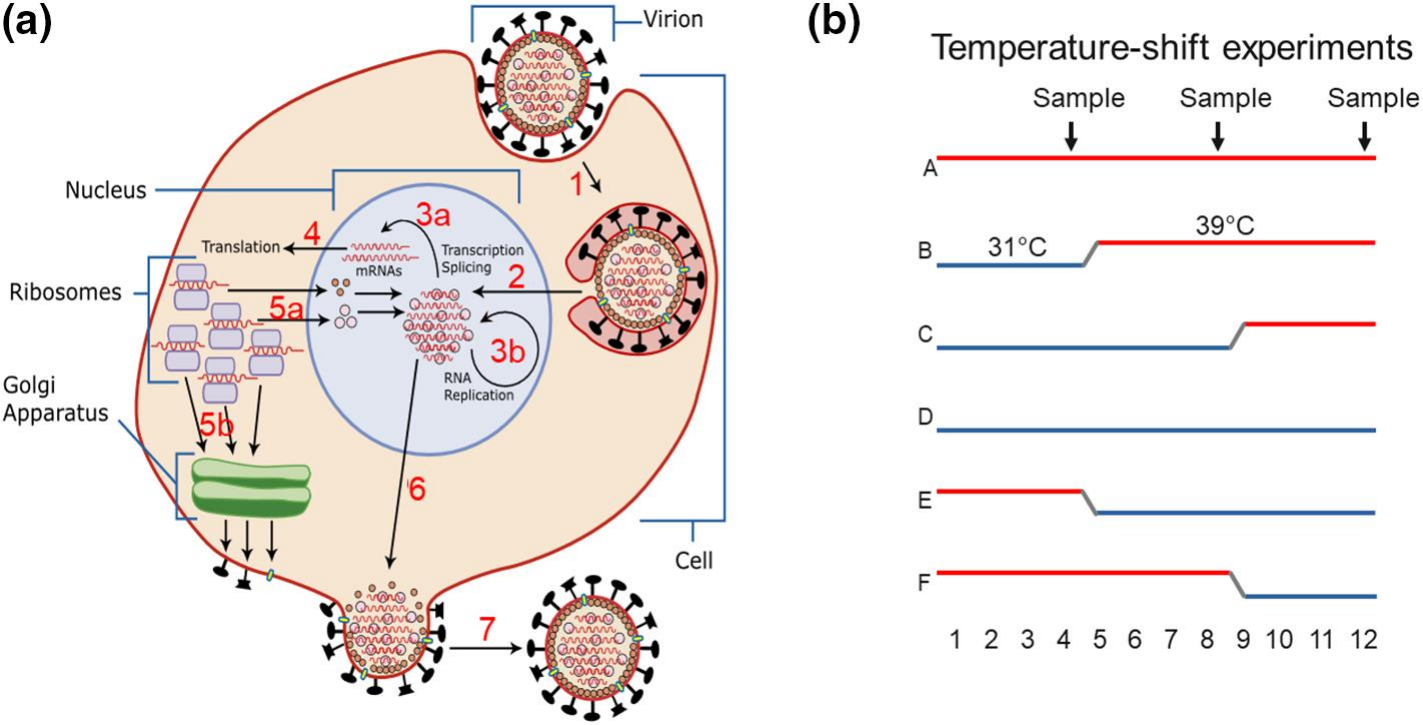
(a) Steps in the life cycle of respiratory viruses that might be thermally sensitive (*ts*). Experiments can determine which steps are *ts* for particular viruses. The diagram shows influenza virus as an example. All numbered steps might be *ts*. The secondary structure of RNA is inherently *ts*, so steps involving RNA are good candidates for *ts* responses. Steps 1, 3 and 4 have been found to be *ts* in influenza in the laboratory (see main text). (b) Temperature shift experiments using cell‐cultures and/or animals can investigate the thermal sensitivity of each step. Samples can be collected periodically (arrows)

The hypothesis explains the four features of seasonal respiratory outbreaks listed above. It is built on four simple premises:

1. Very pathogenic viruses reduce the mobility of their hosts. In well‐established endemic viruses, mild or moderately virulent strains are selected because they are more likely to be transmitted to new hosts than more pathogenic ones.
2. In the case of respiratory viruses, most moderate their pathogenicity by having one or more *ts* steps in their lifecycle (Figure 9(a)), such that they replicate best *below* normal body temperature. Less *ts* strains are more likely to invade the lungs and internal organs and are therefore more virulent. (Note that most biological processes *slow down* at lower temperatures, so this is unusual).
3. This thermal sensitivity confines most respiratory viruses to the nose and throat, where they can cause coughing, sneezing and runny noses, all of which can help to transmit them to other hosts.
4. If ambient temperature remains stable, or rises, *ts* respiratory viruses often remain dormant in the respiratory tracts of their hosts. If ambient temperature drops or hosts are chilled they are likely to become active and may cause respiratory illness.

Based on these premises, this review concludes that the seasonality of viral outbreaks is mainly a consequence of viral thermal sensitivity. Natural selection causes the level of thermal sensitivity to adapt to local conditions. Thermal sensitivity therefore varies with the seasons and in different climates as discussed below.

The Nobel laureate André Lwoff suggested part of the hypothesis in 1959, when he noted that the degree of virulence of viruses is often related to their level of thermal sensitivity.^33^ In 1979, Richman and Murphy developed this further, discussing many examples of thermal sensitivity in natural and lab‐made viral strains, and noting that the near‐universal attenuation of *ts* strains made them good candidates for vaccines.^34^ The full hypothesis was proposed by Shaw Stewart and discussed at length in 2016, focusing on seasonality and the natural selection of strains with varying degrees of thermal sensitivity and pathogenicity.^28^ The same hypothesis was put forward by Eccles in 2020, this time concentrating on the advantages of thermal sensitivity to the virus.^35^ The current shorter review summarises the evidence for TDVT, including recent studies, and discusses its implications for avoiding and treating respiratory illness including Covid‐19.

### 3.1 Premise #1: mild or moderately pathogenic endemic viral strains are more likely to be transmitted

Viral infections that humans occasionally pick up from other animals, normally vertebrates, may cause mild flu‐like symptoms, but they may also be highly pathogenic. For example, at least five unrelated groups of RNA viruses have been identified as causes of human hemorrhagic fevers. These illnesses cause internal or external bleeding and are often fatal. Examples include Whitewater Arroyo virus fever, Rift Valley fever and Lujo virus. The hemorrhagic fevers caused by Marburg virus, Lassa fever virus and Ebola virus have been seen to spread between human hosts.^36^ Other viruses that ‘spilled over’ to humans from other species and caused epidemics with high mortality include Spanish influenza virus, HIV, SARS‐CoV, MERS‐CoV and SARS‐CoV‐2. Clearly these viruses are not yet well‐adapted to their hosts, yet they are much more pathogenic than most well‐established endemic viruses such as cold viruses, supporting the proposal that selective pressures result in a *loss* of virulence as viruses adapt to their hosts.

The moderation of viral pathogenicity is predicted by the ‘transmission‐virulence trade‐off’ hypothesis.^37^ This states that the benefits of increased replication (including increased shedding) must be balanced against the reduction of time during which shedding takes place, and the reduced mobility of hosts. The trade‐off hypothesis was introduced to explain patterns in myxomatosis data.^37^ Myxomatosis has been studied intensely because it provides a classic reference for the rapid evolution of virulence. It is a highly pathogenic viral disease, normally spread by mosquitos, that jumped from New World rabbits to European rabbits. During the first year after its introduction to Australia, it is estimated to have killed 99.5% of the rabbits that it infected.^38^ Within ‘a few years’ genetic resistance to the disease in rabbits increased in some areas, but independent testing with laboratory rabbits showed that milder viral strains emerged more rapidly. For example, a virulent (grade I) strain was introduced to rabbits at Lake Urana in 1952, causing an outbreak with a case‐mortality rate of over 99.5%.^38^ Eleven months after the first outbreak ended, a new outbreak occurred that was caused entirely by attenuated strains of grade III severity. The over‐all trend towards moderate virulence (grade III) can be explained by the selective advantage for mosquito transmission of strains that cause extensive and long‐persisting infectious skin lesions in rabbits.^38^ Interestingly, when mild strains (grade IV) were introduced they often evolved increased virulence, and grade III strains were later recovered. Similar selection can act on respiratory viruses, such that strains with intermediate pathogenicity emerge.

There is other evidence for the moderation of virulence in viral infections. If natural selection tends to reduce the severity of the most pathogenic illnesses such that the period of transmission can increase, there should be differences between illnesses that are transmitted by insects (or other vectors) compared to those transmitted by direct contact. This is because transmission via vectors can take place whether or not the host is immobilised, whereas direct contact between individuals will be reduced by immobilisation. Ewald, who introduced an early version of the trade‐off hypothesis in 1983, showed that pathogens that are transmitted without vectors are significantly more likely to cause illnesses that have mortality below 1% than those transmitted by vectors (*p* < 0.0005).^39^ More virulent pathogens may also be strongly selected in settings such as hospitals and care homes,^40^ where transmission can take place even when individuals are very sick if adequate protective measures are not taken.

### 3.2 Premise #2: most respiratory viruses are *ts*. Less *ts* strains are more virulent, and *vice versa*

Despite practical difficulties in carrying out experiments, viral thermal sensitivity (i.e., viruses replicating faster at temperatures *below* body temperature) has been observed in the wet lab on many occasions. Moreover, if selective pressure is applied in the laboratory and more mutations occur, thermal sensitivity can also be lost or regained in both cells and animal hosts.

It is often easier to propagate respiratory viruses that are freshly collected from patients by incubation at temperatures below 37°C. Table 4 summarises numerous studies that observed optimal temperatures for virus isolation or propagation. In several of these studies, viruses were propagated at one temperature, but found to be more prolific growers at lower temperatures, demonstrating the ts nature of these viruses.^41^, ^42^, ^43^, ^44^, ^45^, ^46^, ^47^, ^48^, ^49^

**TABLE 4:** Studies that reported the optimal temperature for virus isolation or propagation in cell cultures

First author	Ref.	Virus	Host	Temperature conducive to growth	Temperature sub‐optimal for growth	Observation
Price	41	Rhinovirus	Monkey kidney cells	35°C		First isolation of rhinovirus
Tyrrell	42	Three rhinoviruses	Human kidney cells	33°C		Isolation of three rhinoviruses
Bradburne	43	Coronavirus	Human lung fibroblasts	33°C		First isolation of coronavirus
Stern	44	H2N2 ‘Asian’ influenza A	Eggs and monkey kidney cells	33°C	37°C	Viral replication only at low temperature
Stern	44	PR8, FM1 and two more recent H1N1 influenza A strains	Monkey kidney cells	33°C	37°C	One order of magnitude higher titres at the low temperature
Kung	45	H1N1 "Russian” influenza A	Embryonated chicken eggs	33°C	38°C –39°C	9 of 10 strains isolated in China were *ts*
Oxford	46	H1N1 "Russian” influenza A	Canine kidney cells	34°C	38.5°C	17 of 26 strains isolated in Hong Kong, USA, UK, China and USSR were *ts*
Jardon	47	Recombinant adenovirus	Human embryonic kidney cells	34°C	37°C	Three‐fold increase in virus yield at the low temperature
Sato	48	Parainfluenza viruses 1 and 3	Human melanoma cells	34°C		Recommended temperature for virus isolation
Sato	49	Human metapneumovirus	Human melanoma cells	33°C		Recommended temperature for virus isolation

Viral strains can adapt to higher temperatures and lose thermal sensitivity if the right selective pressure is applied in the laboratory. Most laboratory strains of respiratory viruses are propagated in cell cultures at around 37°C, which may result in the rapid loss of *ts* characters, especially since viruses often mutate very rapidly when introduced to new hosts. For example, a study looking specifically at the effect of temperature on the replication of H1N1, H1N2 and H3N2 influenza A viruses found that only 3 of 7 strains examined consistently replicated faster at 37°C than 40°C.^50^ These strains had, however, been propagated at unknown temperatures in the laboratory for up to 23 years, and all strains were ‘amplified’ at 36°C–37°C in eggs for a few days before the study. This procedure might have selected sub‐strains lacking thermal sensitivity. This has been seen in earlier studies ‐ the *ts* character of strains was lost in conditions that allowed rapid replication. Chu et al. passaged a naturally occurring *ts* subclone of the influenza A H3N2 strain Ningxia/11/72 three times through chicken embryos at a low temperature (33°C), and were surprised to find a non‐*ts* strain.^51^ Similarly, Oxford et al. found that when a naturally occurring *ts* virus, A/Eng/116/78 (H1N1), was passaged five times through chicken eggs at 33°C it progressively lost its *ts* character.^46^ Both groups concluded that the *ts* phenotype may confer a selective disadvantage in eggs because eggs allow rapid replication of influenza virus.

The converse trend has been seen: conditions that favour the replication of milder strains have produced *ts* strains. Three reports described the establishment of persistent infections of cell cultures by spontaneously generated *ts* strains of influenza A.^52^, ^53^, ^54^ In a review from 1975,^55^ Preble and Youngner noted that *ts* strains often appear spontaneously in persistent infections of cell cultures with a variety of unrelated insect‐transmitted and respiratory viruses, including Newcastle disease virus, vesicular stomatitis virus, and Sindbis virus. The authors pointed out that a balance between viral and cell replication is required to establish persistent infections and that since *ts* strains tend to be less virulent they may allow such infections to become established.^55^ Similar mechanisms may allow the establishment of persistent infections in animals. For example, foot‐and‐mouth viruses recovered from carrier animals are frequently *ts,* whereas the replication of isolates from animals withacted by temperature.^56^

**FIGURE 10:**
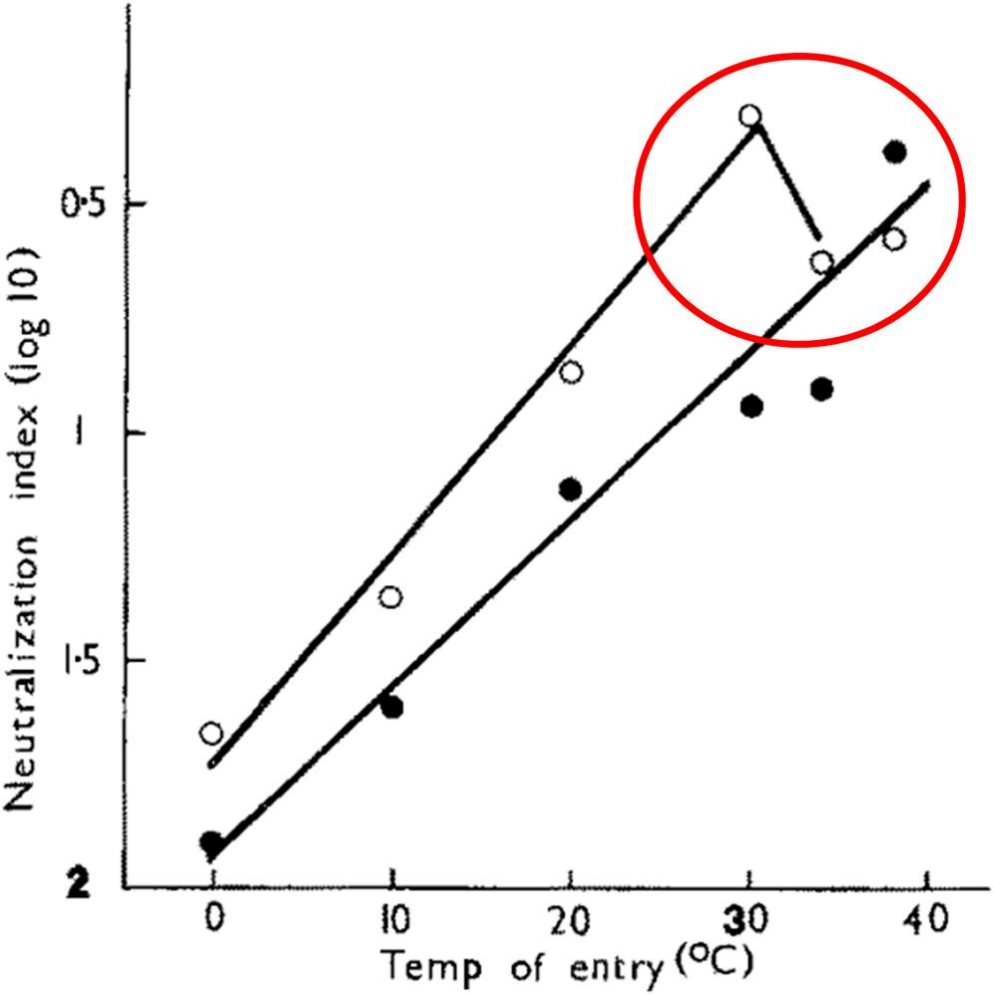
The effect of temperature on influenza virus entry into cells. Virus was preadsorbed to bovine kidney cells at 0°C and then pulsed at varying temperatures of up to 38°C for 120 minutes.^57^ Entry into cells was assessed by escape from subsequent neutralisation using a monoclonal antibody to haemagglutinin. Circles show the triple reassortant virus A/Jap/Bel, black dots the double reassortant A/NIB‐8. Note that at 34°C and 38°C less A/Jap/Bel escaped neutralisation by antibody by entering cells than at 30°C (red oval). This was repeated on two separate occasions using an anti‐H2 serum when 100% of virus escaped neutralisation at 30°C compared to 50% at 38°C (not shown). A/NIB‐8 entry was not *ts* with entry increasing monotonically with temperature. This is one of many reports of unexplained thermal sensitivity (see also Tables 4 and 5). This figure was originally published in Arch Virol 1986;88(3–4):159–66^57^

Table 5 lists a number of laboratory studies that showed that particular steps in the life cycles of viruses were *ts*.^57^, ^58^, ^59^, ^60^, ^61^, ^62^, ^63^, ^64^, ^65^, ^66^, ^67^ For example, Russell found that the entry of a reassortant influenza A virus into cells was *ts* (Figure 10). Virologists tend to focus on mutations that change the *sequences* of viral proteins. However, RNA secondary structure is known to affect protein *expression* and has been shown to control the expression of the proteins NS1 and NEP during influenza A infection.^68^ RNA secondary structure is inherently *ts,* and conserved RNA structures (such as, in SARS‐CoV‐2, the s2m structure, the 3′ UTR pseudoknot, and the coronavirus packaging signal) may comprise ‘RNA thermometers’ contributing to the thermal sensitivity of such viruses.^69^ Two studies found that temperature affects the balance between transcription and viral replication.^64^, ^66^ ‘Silent’ mutations that affect RNA secondary structure may have profound effects on the pathogenicity of respiratory viruses^67^ including SARS‐CoV‐2.

**TABLE 5:** Studies showing that particular steps in the lifecycles of viruses are *ts*

First author	Ref.	Virus	Host/assay system	Temperature conducive to activity	Temperature sub‐optimal for activity	Observation
Fei	58	Influenza H3N1, A/Memphis/71	A glycan‐microarray‐based assay platform	15°C	25°C	The dissociation constant was an order of magnitude higher at the high temperature, implying weaker binding of virus to glycans.
Russell	57	Triple reassortant influenza A	Bovine kidney cells	30°C	38°C	100% of virus entered cells at low temperature, 50% at high (Figure 10).
Takashita	59	Influenza C/Ann Arbor/1/50	Monkey kidney cells	33°C	37°C	Twice as much haemagglutinin‐esterase‐fusion (HEF) protein on the cell surface at low temperature compared to high. Cell fusion at low temperature only. HEF trimer unstable at 37°C.
Plotch	60	Influenza A/WSN/1933	Bovine kidney cells	30°C–32°C		Greatest activity of the viral RNA polymerase.
Muraki	61	Influenza C/Ann Arbor/1/50	“Reverse genetic” system	33°C		Greatest activity of the viral RNA polymerase. “Viral proteins function most efficiently” at this temperature.
Nagele	62	Influenza 3HB t/66	In vitro system	30°C–33°C	40°C	Greatest activity of the viral RNA polymerase at low temperature. At high temperature activity ceased.
Ulmanen	63	Influenza A/WSN/1933	In vitro system	33°C	39.5°C	The fraction of capped RNA primers bound to viral cores at high temperature was unexpectedly reduced relative to the fraction bound at low temperature.
Scholtissek	100	Rostock strain of fowl plague virus	Cytoplasmic extract of infected chick fibroblasts	36°C	41°C	Greatest activity of the viral RNA polymerase was at low temperature. At high temperature the viral RNA polymerase was unstable in vitro and in vivo. The synthesis of haemagglutinin and neuraminidase was unimpaired at high temperature.
Kashiwagi	64	Two H5N1, two H1N1 and one H3N2 influenza A strain	Human embryonic kidney cells expressing various influenza ribonucleoproteins	37°C	42°C	For all strains, viral mRNA increased at high temperature. Genomic RNA (vRNA) production was higher at low temperature.
Ngai	65	Various influenza A strains	Viral RNA polymerase subunits were expressed in human kidney cells	33°C	37°C	2009 pandemic H1N1 RNA polymerase showed significantly higher activity at low temperature. H3N2 showed about 80% higher activity at low temperature, while the highly pathogenic H5N1 strain showed higher activity at high temperature.
Dalton	66	PR8 influenza A/PR/8/34	Human embryonic kidney cells	37°C	42°C	High temperature caused a large decrease in vRNA production and a moderate decrease in cRNA production, but (depending on genome segment) either increased or did not affect mRNA production.
Chursov	67	Wild‐type and cold‐adapted temperature‐sensitive influenza A	Analysis by “a novel in silico method”	32°C	39°C	Areas in the NS2, PA, PB2 and NP mRNAs were predicted to have dissimilar secondary structure (which also varied between wild‐type and cold‐adapted strains) at high and low temperatures.

Abbreviation: HEF, haemagglutinin‐esterase‐fusion; NP, nucleoprotein; vRNA, viral mRNA.

### 3.3 Premise #3: thermal sensitivity confines respiratory viruses to the nose and throat; coughing, sneezing and runny noses encourage transmission

The human respiratory tract is normally colder than most other areas of the body. McFadden et al. showed that there is a temperature gradient that runs from the nostrils (which are close to the temperature of the air being breathed) to the lungs (which are at body temperature).^70^ For example, during quiet breathing of room air the lining of the upper trachea was at 32.0°C, but the subsegmental bronchi were at 35.5°C. The temperature of the respiratory tract fluctuated on each breath, and both breathing colder air and exercise rapidly reduced the temperature.

TDVT suggests that since most respiratory viruses are *ts,* they will not normally replicate in the lungs and internal organs. In 1979 Richman and Murphy pointed out that *ts* influenza, RSV, parainfluenza, and foot‐and‐mouth consistently replicated more rapidly in the nasal cavities of a variety of animals than in their lungs.^34^ Many ‘live’ vaccines use thermal sensitivity to attenuate the strains used, including avoiding lower respiratory tract infections. For example, the FluMist vaccine developed by MedImmune uses a *ts* strain that replicates at 25°C but not body temperature, 37°C–39°C.^71^

**FIGURE 11:**
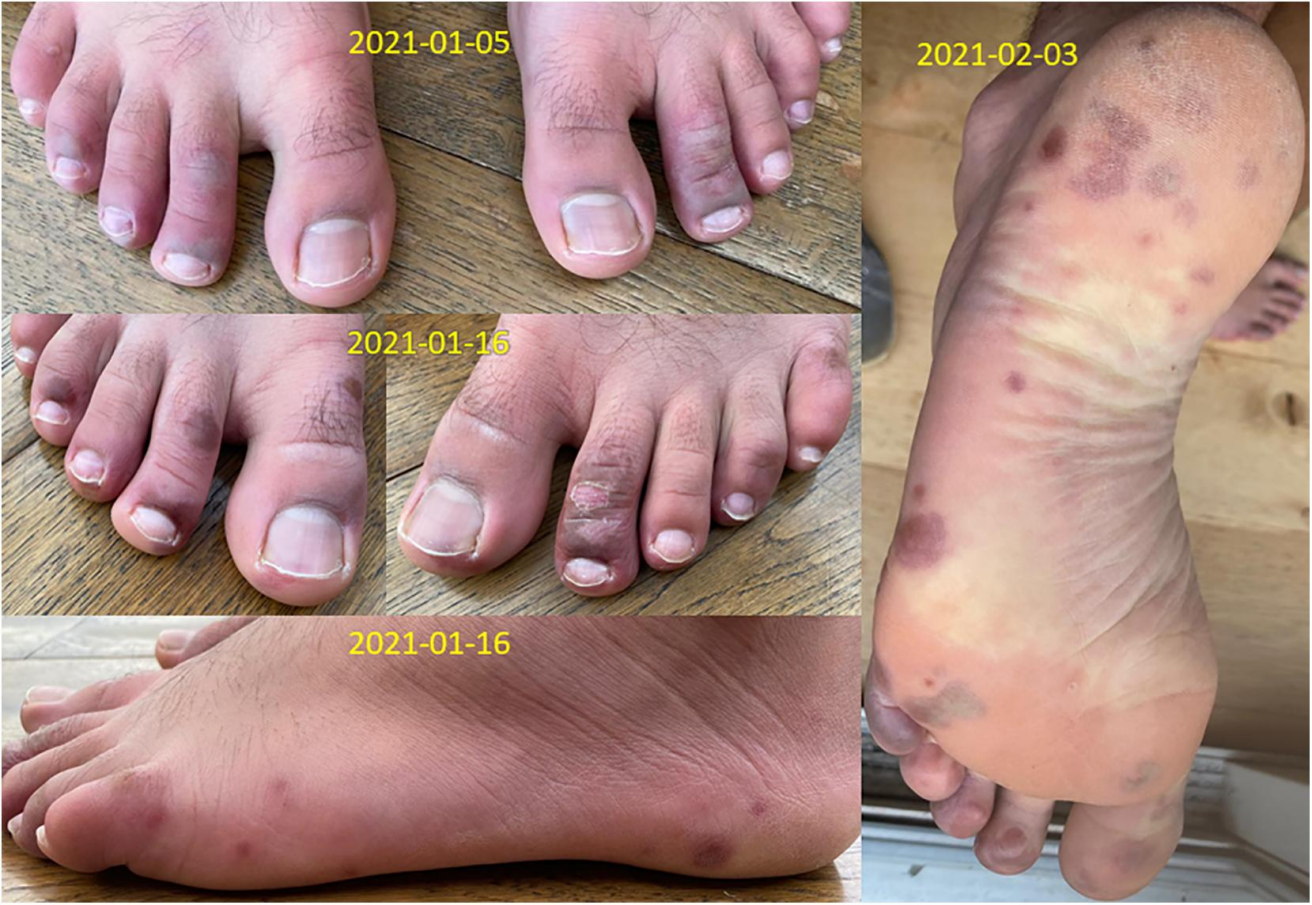
‘Covid toes’ on the feet of a 16‐year‐old boy, who usually did his schoolwork wearing socks without shoes. He felt completely well, but a blood test showed the presence of a viral infection. So‐called Covid toes are blemishes that sometimes appear on the hands and feet, usually pink or purple, after exposure to SARS‐CoV‐2, usually in older children and adolescents. Note the viral‐plaque‐like appearance of blemishes of varying sizes. TDVT suggests that CoV‐2 virions that may reach the bloodstream are more likely to bind to and invade cells in colder parts of the body, possibly causing these visible symptoms

If respiratory viruses enter the bloodstream, TDVT predicts that they may settle and can replicate in other cold tissues such as the skin and the extremities. Virulent human influenza strains occasionally cause rashes. For example, three children who were infected with pandemic H1N1 influenza (‘swine flu’) in 2009 presented with petechial rashes.^72^ Chilblains are normally considered to be an inflammatory skin condition related to ‘an abnormal vascular response to the cold’. They typically present as tender red or bluish lesions located on the dorsal aspect of the fingers, toes, ears and nose.^73^ TDVT suggests that at least some chilblains could be caused by the replication of virus in the extremities. Covid‐19 is associated with chilblain‐like symptoms referred to as ‘Covid toes’ that mainly occur in older children and adolescents, although it has not yet been shown that they are caused by the presence of the virus in the feet. Figure 11 shows plaque‐like blemishes of different sizes on the feet of a 16‐year‐old boy that may possibly represent the sites where individual virions established replication. Covid‐19 also causes a variety of other skin rashes and blisters.^74^ (An alternative explanation is that lung thrombosis might give rise to microemboli in the brain, solid organs and skin^75^).

TDVT also can shed light on the febrile response to infection. The response is well‐preserved across the animal kingdom, and cues delivered by the thermal element of fever are known to stimulate both innate and adaptive immune responses.^76^ It is, however, not clear why raised temperature is used as a protective signal in addition to chemical signals (cytokines and hormones). If, as suggested by TDVT, most respiratory viruses are inhibited by higher temperatures, fever may slow their replication throughout the body. It is interesting that reducing fever by the administration of antipyretics in a randomised prospective study in the ICU was associated with negative outcomes and increased mortality (although the trend was not statistically significant, *p* = 0.06).^77^

### 3.4 Premise #4: *ts* respiratory viruses often remain dormant in the respiratory tracts of their hosts. If ambient temperature drops or hosts are chilled the viruses may become active and cause sickness

Table 6 summarises several studies showing the presence in people of a variety of out‐of‐season or dormant viruses, even in asymptomatic individuals.^78^, ^79^, ^80^, ^81^, ^82^, ^83^, ^84^, ^85^ A large‐scale community study in New York City found that, on average, only 30% of detectable infections caused by 18 common respiratory viruses were symptomatic.^85^ Influenza and metapneumovirus were most pathogenic, with roughly 50% and 70% of cases being symptomatic respectively. Moreover, roughly as many individuals were carrying respiratory viruses in summer as in winter.^84^ Shaman and Galanti found that 50% of infections caused by the endemic coronaviruses within families were asymptomatic.^83^ Table 6 includes studies of respiratory viruses in Antarctica that showed dormancy.^86^, ^87^, ^88^ In one example, parainfluenza shedding occurred throughout the winter isolation period, with two episodes of respiratory illness.^88^ Other studies in the community identified asymptomatic individuals who had not seroconverted, but who were shedding influenza A^78^, ^80^, ^81^ or influenza B.^79^ Thai et al. commented that this ‘may indicate that viral RNA remained in the respiratory tract without being internalised and eliciting an immune response’.^78^

**TABLE 6:** Studies reporting asymptomatic viral infections detected by PCR and/or reactivated by cold conditions

First author	Year	Ref.	Viral species studied	Observation
Galanti	2019	84	Influenza, RSV, parainfluenza, metapneumovirus, rhinovirus, adenovirus, coronavirus	Asymptomatic infection rates exceeded 70% for most viruses, excepting influenza (45%) and metapneumovirus (30%), which produced significantly more severe outcomes.
Galanti	2019	85	Influenza, RSV, parainfluenza, metapneumovirus, rhinovirus, adenovirus, coronavirus	Roughly as many individuals were carrying respiratory viruses in summer as in winter.
Shaman	2020	83	The endemic coronaviruses HKU1, 229E, NL63, and OC43	50% of infections were completely asymptomatic within 9 family clusters.
Granados	2015	82	Human rhinovirus	Asymptomatic rhinovirus activity preceded peak symptomatic activity in September and October and was associated with lower viral load.
Tandale	2010	81	2009 pandemic influenza A (H1N1)	Almost 90% pandemic H1N1 infections were asymptomatic or mild. 18% of PCR‐confirmed cases had not seroconverted.
Papenburg	2010	80	2009 pandemic influenza A (H1N1)	Two asymptomatic cases were detected by RT‐PCR that had not seroconverted.
Thai	2014	78	2009 pandemic influenza A (H1N1)	Four asymptomatically infected contacts had blood collected for serology, of which three had seroconverted.
Foy	1987	79	Influenza B	Of 37 persons shedding virus, 12 were asymptomatic. Of these, 10 did not respond with antibody by any of the five test methods employed.
Cameron	1968	87	Unidentified respiratory virus	Antarctic study. After 12 months of isolation, a respiratory virus was picked up by one individual from a visiting field party (who had already spent more than one month in Antarctica). 17 days after transmission muscle aches and sore throats were experienced by four men after exposure to cold and damp conditions.
Allen	1973	86	Unidentified respiratory virus	Antarctic study. After 17 weeks of complete isolation several men developed colds 7 days after the air temperature fell in 3 days from 0°C to–24°C.
Muchmore	1981	88	Parainfluenza	Antarctic study. Parainfluenza virus was shed by healthy young adults throughout the 8½‐month winter isolation period. Two episodes of respiratory illness caused by parainfluenza occurred after 10 and 29 weeks.

Abbreviations: PCR, polymerase chain reaction; RSV, respiratory syncytial virus.

Since dormant respiratory viruses are often present in the respiratory tracts of otherwise healthy individuals, and since TDVT suggests that such viruses will be activated by local temperature drops, the hypothesis predicts that cold‐weather snaps will cause surges in respiratory disease (Figure 4). In another Antarctic example, several men developed colds after the air temperature fell from 0°C to 24°C.^87^ In 1919, Mudd and Grant, two American doctors, reported that chilling of the body surface (e.g. by placing a wet towel on the back) caused a rapid reduction in the blood flow to the lining of the respiratory tract, together with a rapid fall in its temperature.^89^ This physiological response may cause respiratory sickness after specific chilling events such as wearing wet clothing after rainfall or entering a building kept very cold by air conditioning.^7^

**FIGURE 12:**
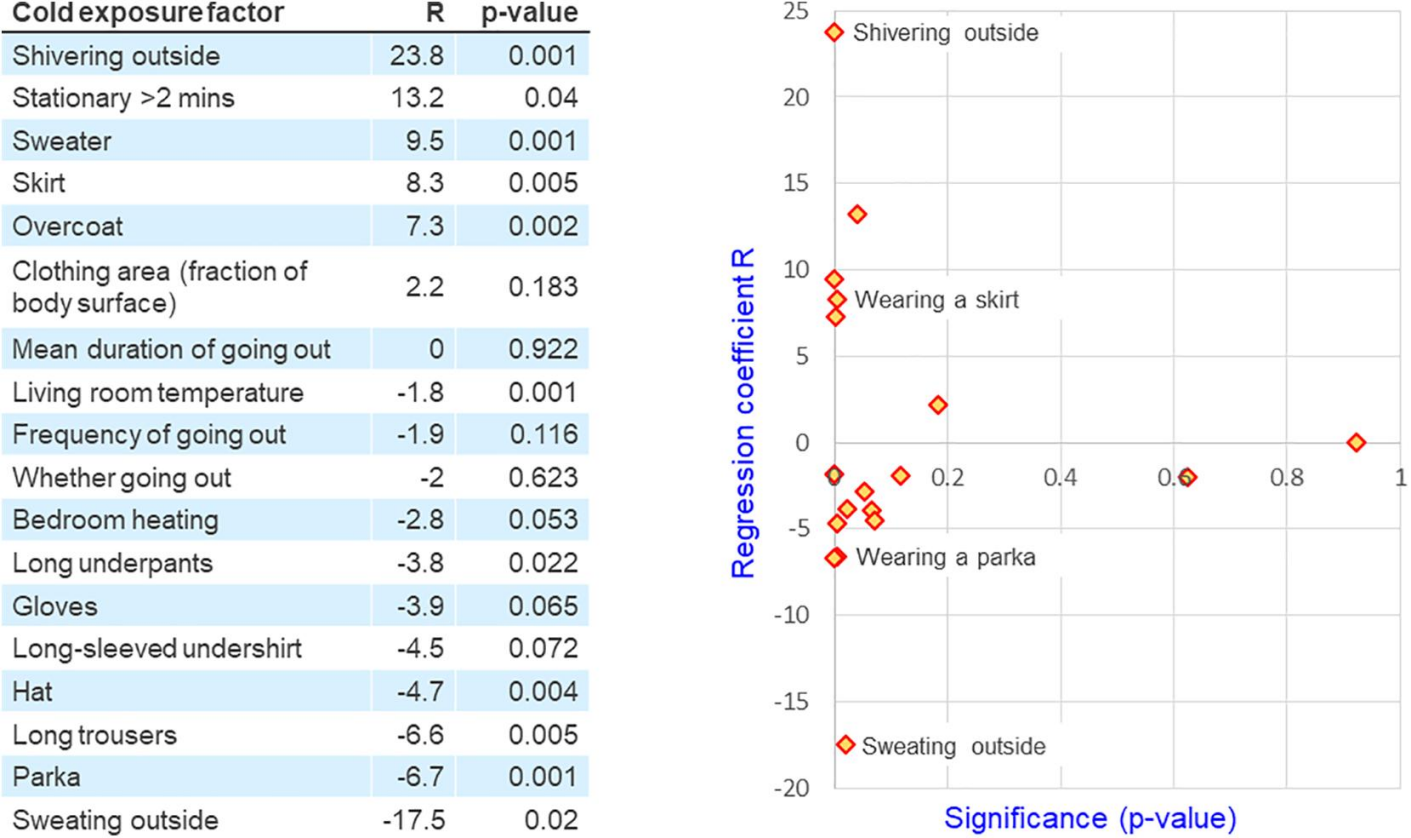
The regression coefficients (R), and their significance (p), for cause‐specific indices of respiratory disease‐related mortality on personal cold‐exposure factors standardised at 7°C mean daily temperature in eight European regions, ranging from northern Finland to Athens.^90^ The Eurowinter Group used market research techniques to analyse the climate and the measures taken to protect individuals from low temperatures. The activities that are most strongly correlated with dying from a respiratory illness are at the top left of the plot, while those that appear to be protective are at the bottom left. Activities that are not related to the chance of death are on the right or at the middle level

A study from 1997 by the Eurowinter Group examined the effect on mortality of exposure to cold. The study found correlations between cold‐exposure factors and death from respiratory illness in seven regions of Europe.^90^ Factors that increased personal chilling such as wearing a skirt (*p* = 0.005), and shivering outside (*p* = 0.001) were significantly correlated with increased risk of death from respiratory illness (Figure 12). Factors that reduced personal chilling such as wearing a hat outside (*p* = 0.004) and outdoor exercise sufficient to cause sweating (*p* = 0.02) were significantly correlated with reduced risk of death from respiratory illness. The finding that protection from viral infection is correlated with outdoor exercise with sweating is particularly interesting. This requires explanation by a combination of TDVT and E3. TDVT says that breathing cold air can ‘wake up’ dormant virions, while E3 says that the lack of chilling, and the good blood flow to the nose and throat (implicit in the stipulation of sweating), may ensure that batches of activated virions ‐ presumably now visible to the immune system ‐ can be destroyed by our defences. Note that the Eurowinter Group found that *frequency of going outside* was not significantly correlated with reduced mortality, suggesting that this apparent protective effect is not solely driven by the regular activation, by cold, of virions in small batches.^90^

Several studies from the 1950s and 1960s, however, have been interpreted as showing that body chilling does not increase the chance that individuals will suffer from respiratory illness (Table 7). Two influential studies by Dowling et al. and Andrewes were performed with volunteers who were inoculated nasally with suspensions of recycled cold viruses (of unknown species) taken from previous volunteers.^22^, ^23^ When individuals were subsequently chilled, the studies found that they were no more likely to get colds than unchilled individuals, and the authors concluded that chilling does not bring on colds. Since these experiments were designed to be carried out within a limited time‐frame, often one week, and infections had to appear quickly if they were to be recorded, it is possible that unusual fast‐acting viral strains that had lost their natural thermal sensitivity were selected over time.^28^ Another study using a type‐15 rhinovirus did find that 16% more of the chilled group developed colds than the unchilled one, but, because the results did not reach statistical significance, the authors reported a ‘failure to demonstrate effect’.^91^ Moreover, the strain used was isolated eight years before the study and grown in WI‐26 cells, a cell‐line that is routinely propagated at 37°C.^92^ It is therefore likely that this strain, like many other laboratory strains, had lost much of its natural thermal sensitivity by the time the study was carried out. Therefore, it is possible that chilling increases the risk of getting a cold caused by a ‘wild’ virus, but not a cold caused by a virus such as was used in these studies.

**TABLE 7:** Studies investigating the effect of chilling on respiratory illness in human volunteers

First author	Year	Ref.	Respiratory virus	Chilled subjects‐ results	Warm subjects‐ results	Comments
Andrewes	1950	22	“Pedigree” (i.e. recycled) virus	Roughly equal numbers of chilled and warm subjects developed colds		The study used 12 chilled and 12 warm subjects
Dowling	1958	23	“Secretions obtained from patients with typical common colds”	88/253 (35%) of those inoculated developed colds	63/175 (36%) of those inoculated developed colds.	Some individuals were not inoculated: 7% of chilled subjects and 16% of warm subjects developed colds
Jackson	1960	113	Specimens from “donors with naturally acquired typical common colds”, or “a virus grown in tissue culture”	Approx. 100/378 (26%) of those inoculated developed colds	Approx. 606/1650 (37%) of those inoculated developed colds	Some individuals were not inoculated: ∼10% of chilled subjects and ∼12% of warm subjects developed colds
Douglas	1968	91	Cultured rhinovirus 15 that was isolated approximately 8 years before the study	4/9 subjects developed colds	2/7 subjects developed colds	Because the numbers were small they were not statistically significant; therefore the authors reported a “failure to demonstrate an effect”
Johnson	2005	101	‘Wild’ viruses that the volunteers happened to be carrying	13/90 subjects developed colds	5/90 subjects developed colds	A significantly greater number of chilled subjects developed colds (*p* = 0.047)

The pattern of dormancy followed by low‐temperature activation may explain the explosive arrival and abrupt termination of influenza epidemics recorded by Hope‐Simpson and others (Figure 7). TDVT suggests that a virus such as influenza can enter a community either without symptoms or with only minor symptoms. If the temperature drops, asymptomatic infections are converted to symptomatic by the activation of dormant viruses. If the temperature then remains stable, no further severe infections develop and the epidemic may end abruptly.

After an initial peak in SARS‐CoV‐2 test positivity in the spring of 2020, rates of illness dramatically decreased during the summer months in most countries situated in temperate locations of the Northern Hemisphere.^93^ SARS‐CoV‐2 is estimated to cause completely asymptomatic infections in 17% of cases.^94^ Case numbers remained low over the summer in many countries, perhaps because asymptomatic individuals were found to be 42% less likely to transmit the virus than symptomatic individuals.^94^ Meanwhile, some areas in countries south of the Equator such as Brazil, Chile and Peru experienced major Covid‐19 epidemics during their cold and/or rainy seasons^d^. Many countries are large enough to have significant regional variations in climate leading to staggered outbreaks. In the autumn, Covid‐19 surged across those countries according to climate, for example regions of the United States with the first cold weather systems saw the first outbreaks.^17^, ^18^, ^19^ (The new cases may have comprised both dormant infections that had been activated by temperature, and new infections transmitted from these newly activated cases.) These observations suggest that despite being a recent ‘spill‐over’ to the human species, SARS‐CoV‐2 has retained or gained significant thermal sensitivity. The Covid‐19 pandemic is an emerging event, however, and additional data will be required to evaluate the effect of thermal sensitivity, as discussed below.

## 4 CONCLUSIONS

### 4.1 Seasonality of respiratory viruses is a result of thermal sensitivity; natural selection based on thermal sensitivity allows respiratory viruses to adapt to local climate conditions

As stated previously, the epidemiology of respiratory viruses still needs rigorous scientific explanations for these observations: (1) in temperate locations colds and flu are seasonal, with a sudden epidemic of colds and flu in the autumn and a rapid response to cold snaps throughout the cold season; (2) roughly the same set of viruses cause respiratory illness all over the world (Table 1), including tropical and temperate locations; and (3) viral outbreaks often begin and end suddenly. Previous explanations assign cause to crowding, enhanced virus survival in the winter, and/or weakened immune systems. However, each of these explanations has flaws when compared to the data on respiratory illnesses.

The four premises of TDVT presented here explain these characteristics of respiratory illness by suggesting that (1) mildly pathogenic endemic viruses are more likely to be transmitted; (2) most respiratory viruses use thermal sensitivity to moderate their pathogenicity; (3) thermal sensitivity helps respiratory viruses stay in the nasal cavity and throat, where they can be transmitted; and (4) viruses are often present but dormant in the nose and throat and can cause sickness if ambient temperature drops or hosts are chilled.

**FIGURE 13:**
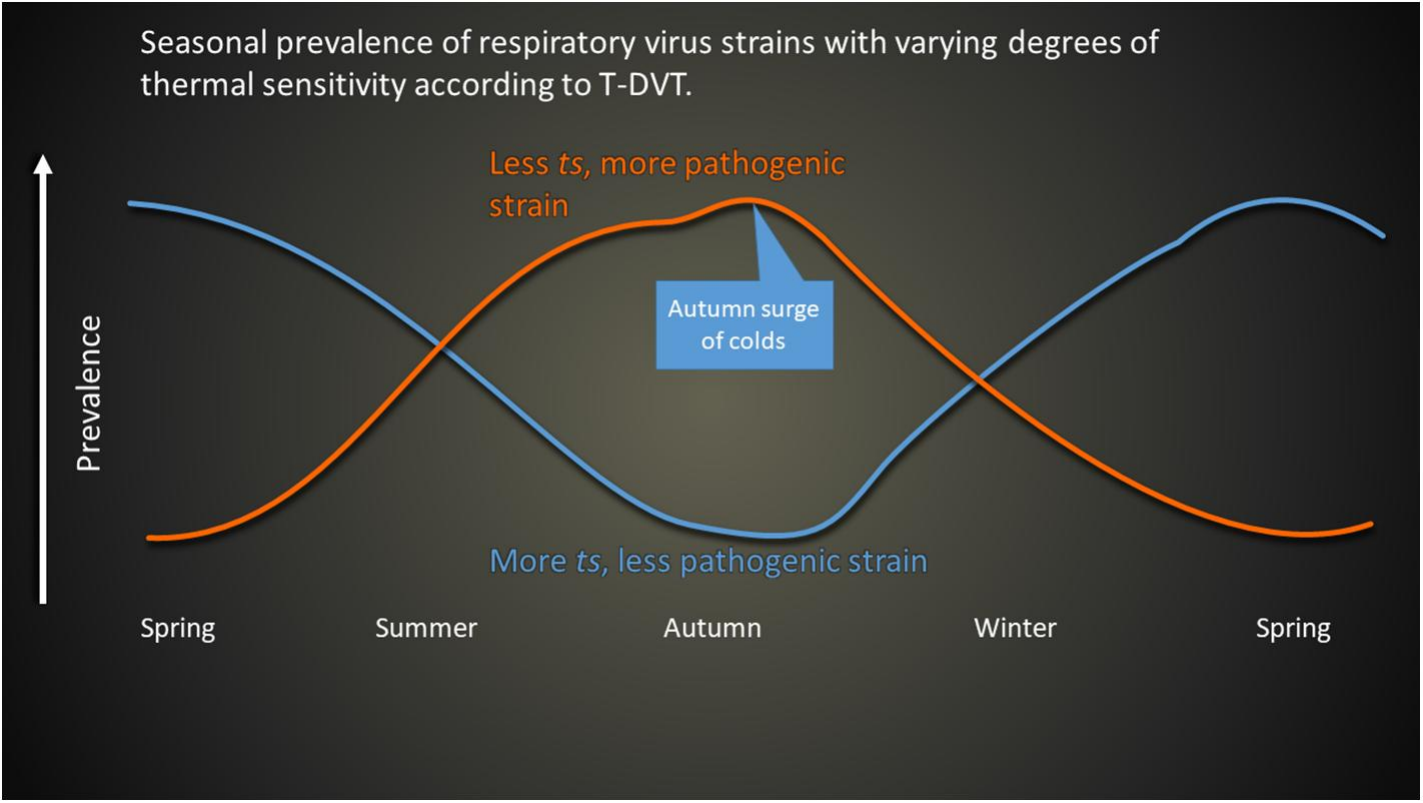
Schematic illustration of the seasonal prevalence in temperate locations of respiratory virus strains with varying degrees of thermal sensitivity, as predicted by TDVT. Two representative strains are shown, one less *ts* (therefore more pathogenic) and one more *ts* (therefore less pathogenic). In reality there would be many strains with varying degrees of thermal sensitivity, and mutations would frequently generate new strains from existing ones with differing thermal properties. During summer the less *ts* strains become more common. During winter, both strains can replicate, but the more *ts* strains increase for a different reason: they are less likely to immobilise their hosts. See the main text for a more detailed description

The autumn surge of respiratory illness in temperate locations (Figure 2) is explained by TDVT by assuming that viruses with a particular level of thermal sensitivity are selected according to the weather/climate, and that this process takes place within a few months. During the hotter weather of summer, the few wild‐type viral strains with low thermal sensitivity are likely to be selected because only less‐*ts* strains are able to replicate and be transmitted at higher temperatures. Other viral strains that are more *ts* may be dormant at this time. (The strains that we refer to as ‘less‐*ts*’ nevertheless retain substantial thermal sensitivity. TDVT suggests that if they were to lose *all* thermal sensitivity they would become highly pathogenic, comparable to the viruses that cause Ebola and other hemorrhagic fevers.) When ambient temperature falls in autumn, the temperature in the nose and throat also falls and the respiratory viruses that are already present quickly become more active, causing an increase in illness and transmission. In winter, the situation changes: the most virulent (less *ts*) strains now immobilise their hosts, so the more *ts* (less virulent) strains are most likely to be transmitted. When ambient temperature rises in the spring, these more *ts* viruses become less active and the number of colds is reduced. The net result is more colds and flu in winter, fewer in summer, with a surge in autumn. Figure 13 shows these trends schematically.

**FIGURE 14:**
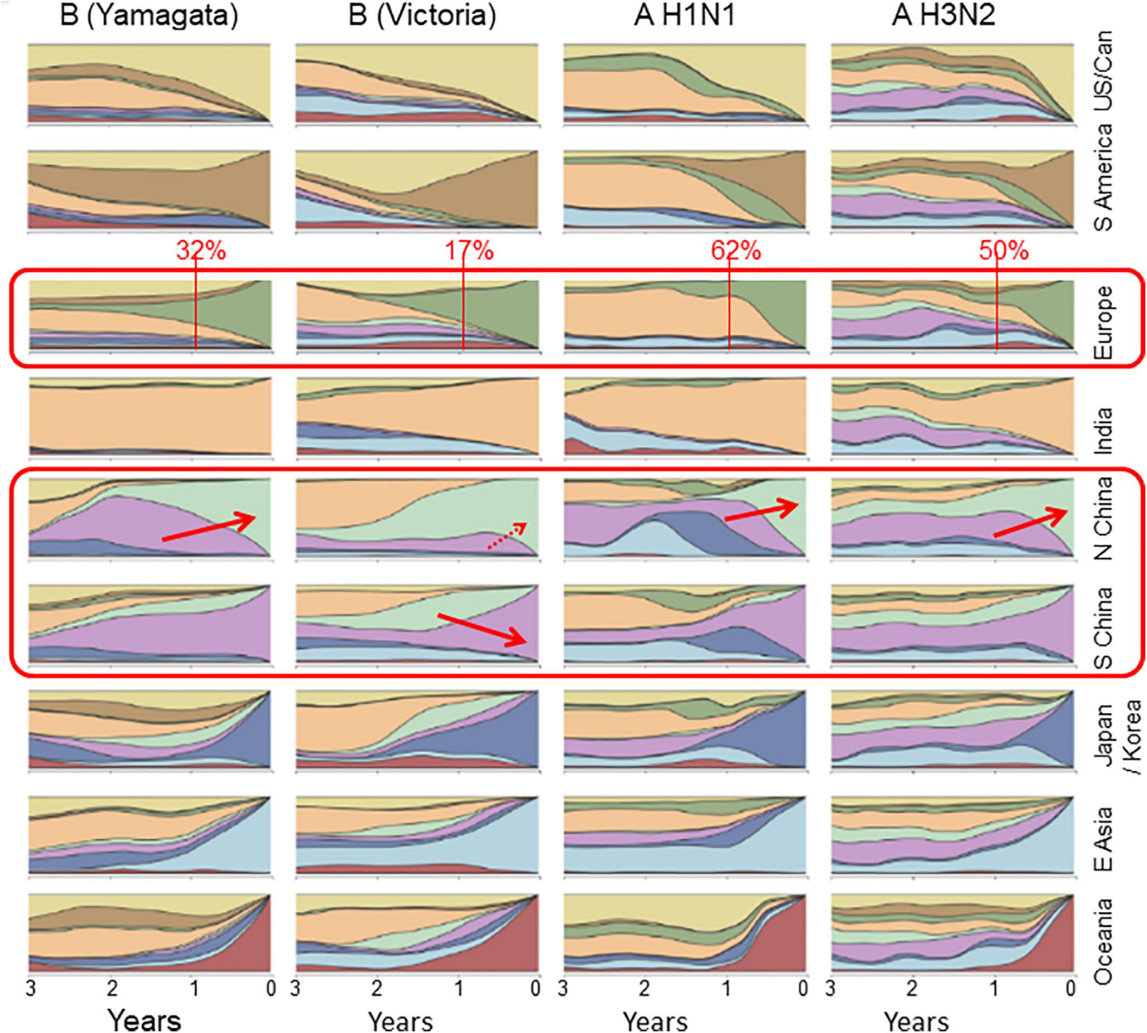
Global movement of influenza strains. The authors divided the world into 9 regions, and, by analysing the sequences of viruses that were sampled worldwide over three years, they charted the movement of influenza strains. The region where each virus was sampled is colour coded, and the colours show where the ancestors of current viruses (in each region) came from. The right‐hand side of each box is only coloured with the region's own colour because strains have had no time to move. As you go to the left the colours show where the ancestral viruses were at the time indicated. For example, ‘Yamagata‐like’ influenza B viruses in India (fourth row, first column) moved very little during the study, with most viruses at the end being descended from strains that were also in India at earlier times. The figure shows that influenza tends to move from hotter locations to colder ones. Many European strains came from India and other hot locations: for example 32% of Yamagata‐like B strains (and 17% of Victoria‐like B strains etc.) that were present in Europe at the end of the study came from strains that were in Tropics or Subtropics one year earlier. In three out of four cases, more strains move from South China to North China than in the opposite direction (indicated by red arrows). European and US strains can also make their way to South China and India, although this movement is less frequent. Adapted from Nature, 2015 Jul;523(7559):217‐20^102^

According to TDVT, respiratory viruses adapt to their local ambient temperature, so we can expect them to spread throughout the world over time and establish reasonably stable equilibria in all locations (albeit disturbed by seasonal temperature fluctuations in temperate locations). Figure 14 shows that influenza strains move freely around the world, but that strains are more likely to move from hotter to colder locations than in the opposite direction. This is predicted by TDVT because tropical strains need to be less *ts* to replicate in nose and throat at higher temperatures, and they are therefore intrinsically more virulent and expected to cause more serious illness if they are transported to temperate locations. Note that the same virus might colonize locations nearer the nose in the Tropics, and nearer the lungs in cold locations.

Studies that attempt to explain the influence of weather, seasonality, and environmental influences on Covid‐19 have yielded ‘contradictory’ and confusing results.^95^ For example, Pan et al. looked at Covid‐19 cases in 202 locations in 8 countries, with matching meteorological data, and found that temperature did not exhibit significant effect on the transmissibility of Covid‐19.^96^ However, Guo et al. looked at meteorological factors and Covid‐19 incidence in 190 countries and concluded that a higher temperature was associated with a lower incidence of Covid‐19.^97^ Ward et al. determined that humidity has a significant effect on SARS‐CoV‐2 spread in Australia,^98^ but Jamshidi et al. did not find that weather was a significant factor.^99^ Note that many meteorological studies examined absolutes of humidity and temperature, not fluctuations. At the start of the pandemic, seasonality may have also been less important because SARS‐CoV‐2 could spread in any climate and season due to lack of immunity. However, as the virus approaches equilibrium, eventually becoming endemic, its thermal sensitivity may increase.

The autumn surge of colds (Figure 2) suggests that natural selection can adjust the thermal sensitivity of respiratory viruses within a few months. Since too high pathogenicity may reduce viral transmission (as patients become bed‐ridden), natural selection may adjust thermal sensitivity to a level that is appropriate to the virus's location, season and climate. These explanations fit the experimental data and observations of viral outbreaks with more rigour than previously offered arguments in favor of crowding, viral survival or immune defences.

## 5 PRACTICAL RECOMMENDATIONS ACCORDING TO TDVT FOR AVOIDING AND TREATING RESPIRATORY ILLNESSES

### 5.1 For avoiding respiratory illness (see Figure 12)

1. Dress warmly to avoid chilling.^90^ Chilling reduces the temperature of the lining of the respiratory tract, allowing dormant respiratory virions to become active and replicate.^89^
2. Avoid standing still outdoors in cold temperatures.^90^
3. Take regular outdoor exercise sufficient to cause sweating.^90^ It seems likely that breathing cold air activates dormant virions ‐ making them visible to the immune system ‐ while dressing warmly improves immune defences in the respiratory tract.^28^ Note that the *combination* of cold air and warm clothing seems to be protective.

### 5.2 For treating existing respiratory illness

1. Keep the temperature of the patient constant and warm.
2. Do not take exercise when symptoms begin. Rapid breathing cools the respiratory tract^70^ and it would be unhelpful to increase the load by activating more virions.
3. Keep the air in the sick‐room warm to increase the blood flow to the respiratory tract.^89^
4. Food and beverages should be at room temperature or warm, not hot.
  - Chilled items may cool the throat and encourage viral replication.
  - Hot drinks are also not recommended. Although viruses often replicate faster at lower temperatures, other viral processes may be activated by warmer temperatures. For example, several studies showed that the production of influenza viral proteins increased at temperatures above the normal temperature of the respiratory tract.^64^, ^66^, ^100^

### 5.3 Future work for the validation of TDVT

The hypothesis is testable at many levels, and suggestions for experiments have already been made.^28^ Important suggestions include:

1. Systematically survey the best temperatures for propagating wild‐type respiratory viruses in tissue cultures.
2. Infect animals with labelled respiratory viruses and determine the positions of replicating viruses in the respiratory tracts of animals that have experienced different temperature regimes (i.e., temperature‐shift time‐course experiments, see Figure 9(b)).
3. Follow viral entry (Figure 10), transcription, splicing, translation, genomic replication, assembly and release of daughter virions from cells in temperature‐shift experiments (Figure 9(b)).
4. Investigate the effect of mutations on thermal sensitivity, including effects on RNA secondary structure, possibly using DNA‐based ‘recombinant’ systems (because DNA sequences are much more stable than the RNA sequences of replicating viruses). Low temperature may directly affect mammalian cell‐lines independently of viral infection, hindering interpretation of experiments. However, the effect of different viral *sequences* can be compared by running them in parallel, allowing clear interpretation of results. Two separate studies of H1N1, H5N1 and H3N2 influenza used this approach (Table 5).^64^, ^65^
5. Run randomised controlled studies to test the recommendations given above for avoiding and treating respiratory illnesses, using for example “wild” respiratory viruses that individuals happen to be carrying during the experiment, following the approach of Johnson and Eccles.^101^

## AUTHOR CONTRIBUTION

Patrick Shaw Stewart and Julia Bach contributed equally to this article. The article is based on a hypothesis that Patrick Shaw Stewart published in 2016.

## Data Availability

Data sharing is not applicable to this article as no new data were created or analysed in this study.

## ENDNOTES

^a^
These explanations were designated M1, M2 and M3 in the 2016 review by Shaw Stewart. They have been changed to E1, E2 and E3 because the previous designations confused some readers. M4 becomes TDVT in this review.

^b^
Google flu search. Retrieved August 2015. Google Flu Trends was a web service operated by Google that attempted to make predictions about influenza activity. After Google ceased publishing Flu Trends in August 2015, the graphical data for related searches was still available as ‘Google flu search’.

^c^
Epidemiological studies show that vitamin D deficiency is associated with increased incidence of cancer and cardiovascular disease, but a very large, randomised, placebo‐controlled trial of supplementation with vitamin D (cholecalciferol) did not result in a lower incidence of either disease. It has been suggested that other important effects of sunshine such as the photorelease of nitric oxides in the skin may explain the link with reduced cardiovascular disease and similar considerations may apply to the incidence of both cancer and respiratory illness.

^d^
During the rainy season in Tropical locations increased humidity reduces evaporation in the respiratory tract and its temperature will be closer to body temperature. When this temperature difference is small any strain that can be transmitted is more likely to infect both the upper and mid (or lower) respiratory tract, causing symptomatic illness.
